# Reversal of Enantioselectivity in the Conjugate Addition Reaction of Cyclic Enones with the CuOTf/Azolium Catalytic System

**DOI:** 10.3390/molecules26113404

**Published:** 2021-06-04

**Authors:** Yuki Nakano, Satoki Shimizu, Chihiro Takeda, Satoshi Sakaguchi

**Affiliations:** Department of Chemistry and Materials Engineering, Faculty of Chemistry, Materials and Bioengineering, Kansai University, Suita, Osaka 564-8680, Japan; k729143@kansai-u.ac.jp (Y.N.); k323441@kansai-u.ac.jp (S.S.); k422297@kansai-u.ac.jp (C.T.)

**Keywords:** asymmetric catalysis, reversal of enantioselectivity, conjugate addition, N-heterocyclic carbene

## Abstract

Hydroxyamide-functionalized azolium salt (NHC•HI **4**) was evaluated for dual enantioselective control in a Cu-catalyzed asymmetric conjugate addition (ACA) reaction. This investigation was based on our previously reported ACA reaction catalyzed using CuOTf combined with NHC•AgI complex **1**. It was revealed that the stereocontrol of the catalytic ACA reaction depended on the order of the addition of the substrates. Additionally, the chiral NHC ligand precursors, substrates, the relationship between the catalyst ee (ee_cat_) and product ee (ee_pro_), and halogen counter anion were completely evaluated. These results suggested that the catalytic performance of the CuOTf/**4** system was comparable with that of the CuOTf/**1** system. Furthermore, to gain knowledge of the Cu species generated using CuOTf and NHC ligand precursor, the reaction of CuOTf with **1** was investigated. Although obtaining the corresponding NHC•CuX species failed, the corresponding NHC•AuCl complex **11** could be synthesized by allowing **1** to react with AuCl•SMe_2_.

## 1. Introduction

The asymmetric conjugate addition (ACA) reaction is a powerful synthetic tool for the stereoselective formation of carbon–carbon bonds [1,2,3,4]. Thus, several chiral ligands for the Cu-catalyzed ACA reaction have been synthesized [5,6,7,8]. However, there is still a need to improve their operational performance with regard to their poor stability and the high cost of their chiral organic components. Therefore, developing a low-cost and high-performance chiral ligand derived from a readily available natural product is highly desirable.

The concept of reversing enantioselectivity has received increasing attention in recent years [9,10,11,12,13,14]. In particular, the development of asymmetric catalytic methods that lead to both enantioenriched products using a single chiral ligand is an important subject in synthetic organic chemistry. This is highlighted when a chiral ligand is synthesized using natural amino acids as a starting material.

In previous studies, we showed that the hydroxyamide-functionalized NHC•AgI (NHC = N-heterocyclic carbene) complex **1b**, derived from leucine, was a versatile chiral ligand precursor for dual enantioselective control in the CuOTf-catalyzed ACA reaction of a cyclic enone with Et_2_Zn [15,16]. The NHC•AgI complex **1** can be easily synthesized by the well-known Ag_2_O method [17,18,19,20,21,22,23,24,25,26,27,28]. Thus, the treatment of azolium salt (NHC•HI, **4**) with a 0.5 equiv. of Ag_2_O afforded the corresponding monodentate NHC•AgI complex **1**. Now, we assumed that the use of **4** in place of **1** in a Cu-catalyzed ACA reaction might provide an alternative method for the switching of enantioselectivity. Here, we decided to investigate the CuOTf-catalyzed ACA reaction under the influence of **4**. Our scope of interest is to study whether the azolium salt **4** has a significant influence on the dual enantioselective control of the catalytic reaction. Additionally, knowledge gained from studies on a Cu species generated from the reaction of CuOTf with the NHC ligand precursor is also reported.

## 2. Results and Discussion

### 2.1. Switching of Enantioselectivity in the Cu-Catalyzed ACA Reaction Using Azolium Iodide

To test the possibility of achieving the switching of enantioselectivity, 2-cyclohexen-1-one (**2**) was allowed to react with Et_2_Zn catalyzed by CuOTf/**4b** (R^1^ = Et, R^2^ = Bn) (Table 1). To compare the relative abilities between NHC•HI **4** and NHC•AgI **1**, the results of the reaction employing **1b** are also listed in Table 1 as Entry 1. Under the exact same reaction conditions, the enantioselectivity was switched by changing the order of addition of the substrates in the CuOTf/**1** catalytic system, although low product yield was observed [15]. 

To a THF solution containing CuOTf (6 mol%), **4b** (4 mol%), and **2** was added 3 equiv. of Et_2_Zn, and the mixture was then allowed to react for three hours (Method A). This catalytic reaction produced (*R*)-3-ethylcyclohexanone ((*R*)-**3**) in 91% yield with 60% ee (Table 1, Entry 2). When **2** was added as the last component to the mixture of CuOTf (4 mol%), **4b** (10 mol%), and Et_2_Zn in THF (Method B), the conjugate adduct ((*S*)-**3**) with the opposite configuration was obtained in 91% yield with 80% ee (Entry 2). These results in the ACA reactions with the CuOTf/**4** system were comparable with those obtained in the reactions with the CuOTf/**1** system. Thus, the CuOTf/**4** catalytic system can provide an alternative, simplified reaction procedure for the switching of enantioselectivity in an ACA reaction. This method offers an important advantage to avoid preparation of NHC•AgI complex.

Next, various azolium iodides derived using commercially available β-amino alcohols were evaluated for the dual enantioselective control of the catalytic ACA reaction. Table 2 summarizes the results of the ACA reactions by Method A (left column) and Method B (right column).

In the ACA reaction by Method A, the introduction of a methyl substituent in place of a benzyl substituent into the NHC ring, far from the stereogenic center of the chiral ligand, significantly decreased the stereoselectivity of the catalytic reaction (Entries 1–5 vs. Entries 6–10, left column). The increase in the steric demand of the alkyl substituent R^1^ on the chiral ligand side-arm led to a high stereoselectivity. Thus, *sec*-butyl and *tert*-butyl groups work more efficiently to give (*R*)-**3** with 71% and 75% ee, respectively (Entries 4 and 5, left column).

A different tendency with respect to the stereodirecting group on the ligand was observed in the reaction by Method B in comparison with Method A (Table 2, right column). A poor product ((*S*)-**3**) yield and ee were obtained when **2** was allowed to react with Et_2_Zn catalyzed using CuOTf combined with **4d** (R^1^ = *^s^*Bu) (Entry 4). No reversal of enantioselectivity was achieved in the ACA reaction with **4e** (R^1^ = *^t^*Bu) under these reaction conditions (Entry 5). Similar observations were reported in the ACA reaction with the CuOTf/NHC•AgI **1** catalytic system. Better performances were obtained when the ACA reaction was conducted using chiral azolium salt with ethyl or isobutyl groups. Thus, **4c**, **5b,** and **5c** provided (*S*)-**3** with 87%, 89%, and 86% ee, respectively (Entries 3, 7, and 8).

Table 3 summarizes the dual enantioselective control in the ACA reactions of several cyclic enones with dialkylzinc by Methods A and B. We chose the CuOTf/**4b** catalytic system for the switching of enantioselectivity.

First, azolium salt, *ent*-**4b**, which has the opposite configuration to **4b**, was synthesized from (*R*)-2-amino-1-butanol. As expected, when Et_2_Zn was added to a THF solution of CuOTf, *ent*-**4b**, and **2** (Method A), the corresponding 1,4-adduct, (*S*)-**3**, was preferentially obtained in 79% yield with 69% ee (Entry 2). In contrast, when Et_2_Zn was added first followed by **2** (Method B), (*R*)-**3** was obtained as the major product (Entry 2). Dual enantioselective control was also observed in the reactions of 4,4-dimethyl-2-cyclohexen-1-one with Et_2_Zn to afford 3-ethyl-4,4-dimethylcyclohexanone (**6**), although somewhat long reaction times were needed (Entry 3). A seven-membered cyclic enone, 2-cyclohepten-1-one, was also evaluated. The ACA reactions catalyzed using the CuOTf/**4b** system under the standard reaction conditions proceeded smoothly to afford 3-ethylcycloheptanone (**7**) (Entryy 4). Thus, (*R*)-**7** was obtained in 89% yield with 73% ee in the reaction by Method A, whereas an inversion of enantioselectivity was induced by Method B, affording (*S*)-**7** in 84% yield with 81% ee.

An attempt to use Me_2_Zn in the place of Et_2_Zn for the ACA reactions using Method A failed, probably owing to the low nucleophilicity of the alkylating reagent. However, the ACA reactions by Method B did take place to give the desired 1,4-adducts (Entries 5 and 6). An excellent ee value (93%) was obtained in the reaction of **2** with Me_2_Zn by Method B (Entry 5). Similarly, the ACA reaction of 2-cyclohepten-1-one with Me_2_Zn by Method B afforded (*S*)-3-methylcycloheptanone ((*S*)-**9**) in 90% yield and with 93% ee (Entry 6).

### 2.2. Influence of Counter Anion on Azolium Salt: The Effect of Halide Ion

Encouraged by the success with the reversal of enantioselectivity using azolium iodide (NHC•HI, **4b**) as a key chiral ligand, our interest turned to the ACA reaction using the CuOTf/azolium bromide (NHC•HBr, **10b**) catalytic system. The purpose of this study is to obtain an insight into various aspects of the effect of a halide ion.

**Scheme 1:**
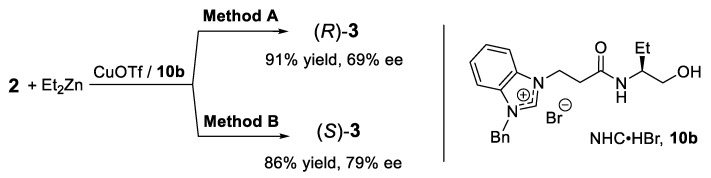
Switching of stereoselectivity using NHC∙HBr (**10b**).

In a similar manner to the CuOTf/**4b** catalytic system, the stereocontrol of the ACA reaction using **10b** depended on the order of the addition of the substrates (Scheme 1). Et_2_Zn was added to THF solution containing CuOTf (6 mol%), **10b** (4 mol%), and **2** to yield (*R*)-**3** in 91% yield and 69% ee (Method A). When **2** was added as the last component to a mixture of CuOTf (4 mol%), **10b** (10 mol%), and Et_2_Zn in THF (Method B), the ACA reaction afforded (*S*)-**3** with 79% ee (Scheme 1).

In the past two decades, a huge variety of chiral ligands have been developed for Cu-catalyzed ACA reactions. Investigation of the relationship between the optical purities of the chiral ligand and product can help explain reaction mechanisms [29,30,31,32]. There are many reports on the observation of nonlinear effects in ACA reactions. Therefore, we next studied the relationship between the catalyst ee (ee_cat_) and product ee (ee_pro_) in the Cu-catalyzed ACA reaction under the influence of azolium iodide (**4b**) or azolium bromide (**10b**).

**Figure 1:**
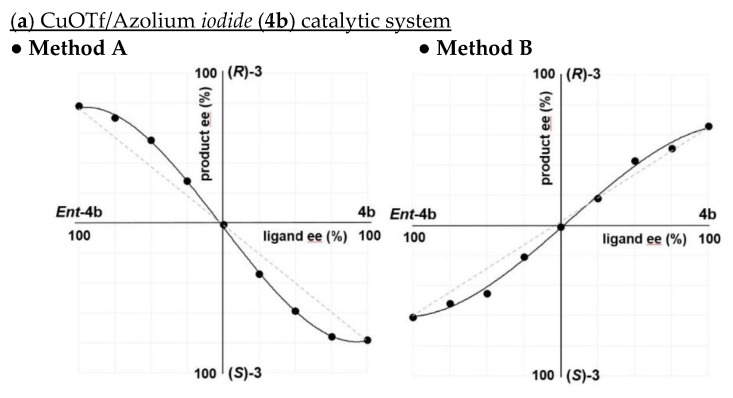

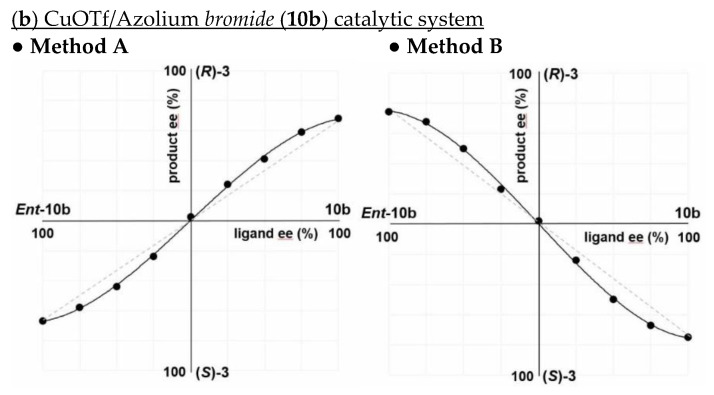
Chiral amplification phenomenon in reaction of **2** with Et_2_Zn by methods **A** and **B**.

Various mixtures of **4b** (or **10b**) and *ent*-**4b** (or *ent*-**10b**) were carefully prepared. The results of the ACA reactions in both asymmetric reaction systems (Methods A and B) are summarized in Figure 1. The ACA reaction catalyzed by the CuOTf/**4b** system by Method A provided sufficient chiral amplification to reach an enantiopure end state (Figure 1a). In the reaction by Method B, a nonlinear effect was also observed. These results probably arise from the presence of di(oligo)meric species. Moreover, in the ACA reaction using the CuOTf/**10b** catalytic system by Method A or B, a similar chiral amplification phenomenon was observed (Figure 1b). As a result, it can be concluded that the halide ion on the chiral azolium salt did not dramatically affect the catalytic ACA reaction.

**Scheme 2:**
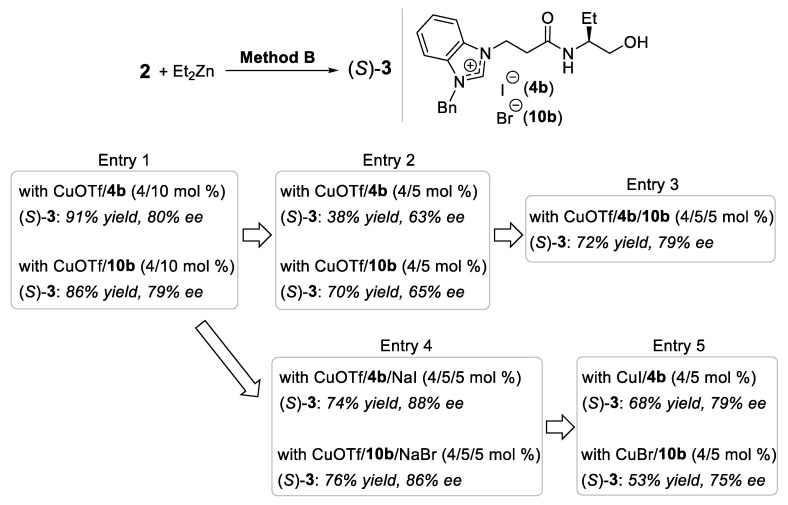
Effect of halide anion (I^-^ vs. Br^-^) in Method **B** to afford (*S*)-**3**.

As mentioned above, the ee value obtained in the ACA reaction by Method B was superior to that obtained in the ACA reaction by Method A. A successful result was obtained when the ACA reaction by Method B was carried out in the presence of CuOTf (4 mol%) and azolium salt (10 mol%, **4b** or **10b**). Next, we investigated why an excess amount of azolium salt is needed (Scheme 2).

A decrease in the amount of the chiral ligand (**4b** or **10b**) showed a significant influence on the catalytic ACA reaction (Entry 1 vs. Entry 2). For example, the reaction of **2** with Et_2_Zn catalyzed using the CuOTf/**4b** (4/5 mol%) system afforded (*S*)-**3** in only 38% yield with 63% ee (Entry 2). When the ACA reaction was conducted with CuOTf/**4b/10b** (4/5/5 mol%), the desired product was obtained in 72% yield with 79% ee (Entry 3). This result might indicate that a bis(NHC)-Cu species is generated under these reaction conditions.

On the other hand, it was also assumed that an excess amount of the azolium salt is needed to supply a halide species. Notably, the ACA reaction under the influence of the CuOTf/**4b/**NaI (4/5/5 mol%) system yielded (*S*)-**3** in 74% yield with 88% ee (Entry 4). Similarly, the CuOTf/**10b/**NaBr (4/5/5 mol%) system was also effective (Entry 4). These results might suggest that the success of the ACA reaction by Method B requires a Cu/azolium/halide catalytic ratio of 1/1/2. Indeed, (*S*)-**3** was obtained with satisfactory enantioselectivity in the reaction of **2** with Et_2_Zn catalyzed by CuI/**4b** (4/5 mol%) or CuBr/**10b** (4/5 mol%), although the yield of (*S*)-**3** was somewhat lowered (Entry 5).

### 2.3. Investigation of the Reaction of NHC•AgI **1b** with CuOTf

**Scheme 3:**
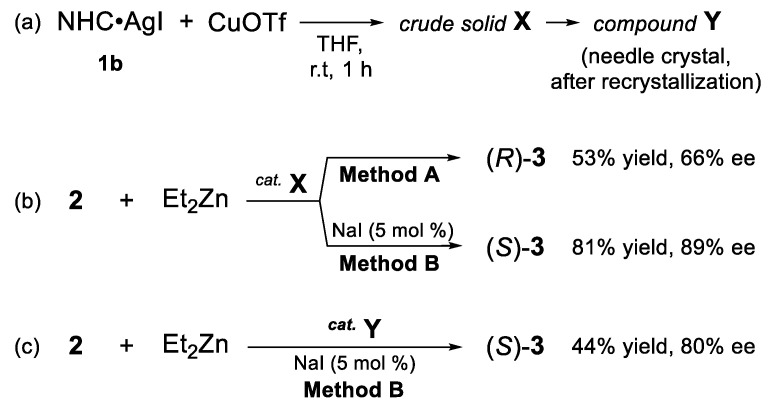
(**a**) Investigation of the reaction of **1b** and CuOTf. (**b**) catalytic activity of the crude solid product **X**. (**c**) catalytic activity of the purified product **Y**.

As mentioned in the introductory section, the well-defined NHC•AgI **1b** was synthesized by the Ag_2_O method. Then, the catalytic ACA reaction catalyzed by CuOTf in combination with **1b** was achieved. Next, our interest turned to the chemical species obtained from the reaction of **1b** with CuOTf. Scheme 3 summarizes the investigation on the reaction of **1b** with CuOTf.

A 1:1 mixture of **1b** (0.05 mmol, 29 mg) and CuOTf•1/2C_6_H_6_ (0.05 mmol, 13 mg) was stirred in THF at room temperature. During this reaction, the appearance of a yellowish-brown solid in a clear pale blue solution was observed. After 1 h, the precipitate was filtered with suction, and then the resulting filtrate was evaporated to dryness in vacuo to afford 24 mg of crude solid **X** (Scheme 3a). At this stage, our scope of interest was to study whether the solid **X** obtained acts as a catalyst for the ACA reaction. Thus, **2** (1 mmol) was reacted with 3 equiv. of Et_2_Zn in the presence of 24 mg of **X**. By Method A, the desired 1,4-adduct, (*R*)-**3**, was produced in 53% yield with 66% ee. In contrast, Method B furnished (*S*)-**3** in 81% yield with 89% ee in the presence of 5 mol% of NaI (Scheme 3b). These results strongly indicated that a catalytically active species that realizes the switching of enantioselectivity in the ACA reaction can be obtained from the independent reaction of **1b** with CuOTf. In addition, it is worth noting that solid **X** is very stable to air and moisture, and solid **X** is easy to store without any special precautions.

Next, the purification of the crude solid product **X** and catalytic activity of the purified product **Y**, obtained from solid **X**, were investigated (Scheme 3a,c). After the reaction of **1b** (0.1 mmol, 59 mg) with CuOTf•1/2C_6_H_6_ (0.1 mmol, 25 mg) in THF at room temperature for 1 h, the crude solid product **X** (56 mg) was obtained according to the above-mentioned procedure. Then, solid **X** (56 mg) was purified by reprecipitation using THF and Et_2_O to afford a whitish blue-green solid. Then, 28 mg of whitish blue-green needle crystals of the purified product **Y** were obtained by layering solutions of the resulting whitish blue-green solid in THF with Et_2_O and allowing slow diffusion at room temperature (Scheme 3a and Figure S1). The performance of product **Y** in the ACA reaction by Method B was investigated. Treatment of **2** (1 mmol) with Et_2_Zn in the presence of **Y** (24 mg) and NaI (5 mol%) afforded (*S*)-**3** in 44% yield with 80% ee (Scheme 3c). This indicated that the purified product **Y** still involves a catalytically active species, although a somewhat lower yield and enantioselectivity were observed in comparison with the crude solid product **X**.

Figure S1 shows the ^1^H NMR spectra of the product **Y** in DMSO-*d*_6_. The spectra of NHC•AgI **1b** and NHC•HI **4b** are also shown to compare the differences between **Y** and these starting materials (Figure S1). In the ^1^H NMR spectrum of **4b**, a signal at δ 9.9 ppm, which is attributed to the proton in the C_2_ position of **4b** appeared (Figure S1c). However, that signal was not observed in the ^1^H NMR spectrum of **1b** because deprotonation occurred in the synthesis of **1b** from the reaction of **4b** with Ag_2_O (Figure S1b). It was found that a signal at δ 9.7 ppm was newly observed in the ^1^H NMR spectra of **Y** (Figure S1a). Additionally, in ^13^C NMR spectra of **1b**, the characteristic carbene C signal at δ191 ppm was observed in **1b**. Disappearance of the signal at δ 191 ppm and appearance of a signal at δ 135 ppm were observed in the ^13^C NMR spectrum of **Y**. Overall, the NMR spectrum of **Y** was quite similar to that of NHC•HI **4b**. These results strongly indicate that **Y** contains the same azolium cation (NHC•H^+^) as **4b**. Recently, Ollevier observed that treatment of a NHC•CuX complex with atmospheric air in CH_2_Cl_2_ afforded the corresponding hydrolysis product such as an azolium compound [33,34].

**Scheme 4:**
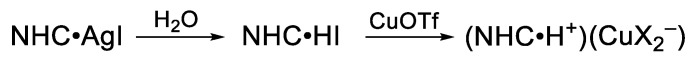
A plausible reaction pathway.

As shown in Scheme 3, product **Y** catalyzed the ACA reaction of **2** with Et_2_Zn. In contrast, almost no reaction was observed in the ACA reaction of **2** with Et_2_Zn in the presence of **4b** without CuOTf precatalyst. In addition, the melting point of **Y** (145.0 °C–145.5 °C) differs from that of **4b** (104.6 °C–105.3 °C). These results indicate that **Y** contains Cu species and that **Y** is not exactly the same as NHC•HI **4b**. Unfortunately, the purified product **Y** failed to yield satisfactory crystals for an X-ray crystal structure. Although the identification of **Y** was difficult at this stage, we speculated that **Y** might consist of an azolium cation (NHC•H^+^) and a cuprate(I) anion (CuX_2_^–^) (Scheme 4). It was assumed that the azolium species (NHC•HI) would be generated by the hydrolysis of NHC•AgI **1b**. This is similar to Ollevier’s observation [33,34]. Subsequently, the resulting NHC•HI would react with CuOTf to afford the product **Y** [(NHC•H^+^)(CuX_2_^–^)] (X=I and/or OTf) (Scheme 4). This might explain why almost the same results were obtained in the Cu-catalyzed ACA reaction under the influence of NHC•HI (the present work) in comparison with the ACA reaction under the NHC•AgI complex (the previous work).

**Scheme 5:**
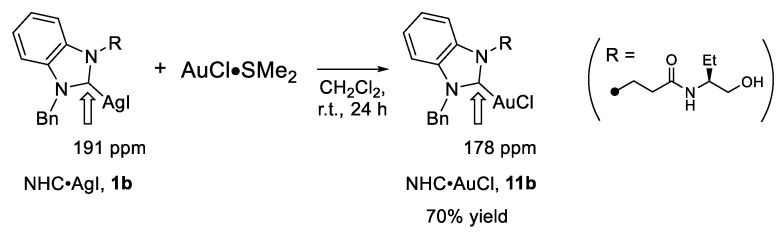
Reaction of **1b** with AuCl.

Finally, we investigated the ligand transfer reaction between NHC•AgI **1b** and Au species to form an NHC•AuX complex (Scheme 5). As shown in Scheme 3, the reaction **1b** with CuOTf did not provide the corresponding NHC•CuX complex desired. However, it was found that the corresponding NHC•AuX complex could be synthesized with ease when 0.10 mmol of **1b** was allowed to react with 0.11 mmol of AuCl•SMe_2_ in CH_2_Cl_2_ at room temperature for 24 h. This reaction yielded the desired NHC•AuCl complex **11b**, whose structure was confirmed with ^1^H and ^13^C NMR spectroscopy and elemental analysis. The carbene C signal at δ 178 ppm was observed in NHC•AuCl **11b**, whereas, the carbene C signal of NHC•AgI **1b** was observed at δ 191 ppm. The assignment of the characteristic carbene C atom in **11b** could be made based on a comparison with those reported for NHC-Au complexes [35,36,37].

## 3. Materials and Methods

### 3.1. General Procedures

Et_2_Zn and Me_2_Zn were purchased from Sigma-Aldrich, St. Louis, MO, USA and used without further purification. Dry THF was purchased from FUJIFILM Wako Pure Chemical Corporation, Osaka, Japan. All other chemical reagents and solvents were obtained using commercial sources. Column chromatography was performed using silica gel 60 (63–210 μm) purchased from KANTO CHEMICAL CO., INC. Tokyo, Japan. ^1^H NMR spectra were recorded using a JEOL ECA400 (400 MHz for ^1^H NMR and 100 MHz for ^13^C NMR) spectrometer (JEOL Ltd. Warszawa, Poland) (see Supplementary Materials). Chemical shifts were reported downfield from TMS (δ = 0 ppm) for ^1^H NMR. For ^13^C NMR, chemical shifts were reported on the scale relative to the solvent used as an internal reference. Elemental analyses were performed at Osaka University, Osaka, Japan. Enantiomeric excesses were measured by gas chromatography. Azolium salts were synthesized from the corresponding azole and benzyl halide (or methyl iodide) according to our previously reported procedure [15,16,38].

### 3.2. General Procedure for Method A

The reaction was performed under argon atmosphere. A flask under argon atmosphere, was charged with CuOTf•1/2C_6_H_6_ (15 mg, 0.06 mmol) and **4b** (19 mg, 0.04 mmol). Then, a solution of enone **3** (96 mg, 1 mmol) in anhydrous THF (9 mL) was added. The resulting mixture was stirred at room temperature for 1 h. After the mixture was cooled to −20 °C, a solution of Et_2_Zn (3 mmol, 1 M in hexanes, 3 mL) was added dropwise over a period of 10 min. The reaction mixture was stirred at −20 °C for 3 h. The reaction was quenched by adding 10% aq. HCl. The resulting mixture was extracted using diisopropyl ether and dried over Na_2_SO_4_. The product was purified by silica gel column chromatography with a mixture of hexane/EtOAc.

### 3.3. General Procedure for Method B

The reaction was performed under open-air conditions. CuOTf•1/2C_6_H_6_ (10 mg, 0.04 mmol) and **4b** (48 mg, 0.10 mmol) were added to anhydrous THF (5.5 mL). After stirring at room temperature for 1 h, the mixture was cooled to 0 °C. Then, Et_2_Zn (3 mmol, 1 M in hexanes, 3 mL) was added to the reaction vessel. After the resulting mixture was stirred at room temperature for 30 min, a solution of enone **3** (96 mg, 1 mmol) in anhydrous THF (1.5 mL) was added dropwise over a period of 10 min. The reaction mixture was stirred at room temperature for 3 h.

### 3.4. Procedure for Reaction of NHC•AgI Complex 1b with CuOTf•1/2C_6_H_6_

The reaction mixture of NHC•AgI complex **1b** (0.10 mmol, 59 mg) and CuOTf•1/2C_6_H_6_ (0.10 mmol, 25 mg) in THF (9 mL) was stirred at room temperature for 1 h under open-air conditions. After the filtration of the reaction mixture, the filtrate (light blue-green solution) was evaporated to dryness in vacuo to afford 56 mg of whitish blue-green solid (crude product **X**). Needle crystals (28 mg, product **Y**) were obtained by layering solutions of **X** in THF with Et_2_O and allowing slow diffusion at room temperature. The following analytical data (NMR and elem. analysis) are given for product **Y**. Elemental analysis might indicate that product **Y** is a Cu complex consist of azolium iodide and CuOTf.

^1^H-NMR (DMSO-*d*_6_, 400 MHz): δ 9.85 (s, 1H), 8.09 (d, *J* = 8.2 Hz, 1H), 7.92 (d, *J* = 8.2 Hz, 1H), 7.74 (d, *J* = 8.2 Hz, 1H), 7.73–7.54 (m, 2H), 7.48–7.43 (m, 2H), 7.40–7.33 (m, 3H), 5.77 (s, 2H), 4.72–4.68 (m, 2H), 4.58–4.51 (m, 1H), 3.57–3.49 (m, 1H), 3.31–3.21 (m, 2H), 2.81–3.72 (m, 2H), 1.46–1.41 (m, 1H), 1.28–1.15 (m, 1H), 0.53 (t, *J* = 7.3 Hz, 3H); ^13^C-NMR (DMSO-*d*_6_, 100 MHz): δ 169.3, 141.8, 132.2, 131.1, 130.9, 129.5, 129.3, 128.3, 128.3, 127.3, 118.8, 113.3, 63.3, 52.8, 49.8, 43.1, 34.4, 23.1, 9.8. Anal. Calc. for (C_21_H_26_IN_3_O_2_)_10_•(CCuF_3_O_3_S): C, 50.62; H, 5.24; N, 8.39. Found: C, 50.99; H, 5.38 N, 8.10%. M.p. 145.0–145.5 °C.

### 3.5. Procedure for Synthesis of NHC•AuCl Complex 1

The reaction mixture of NHC•AgI complex **1b** (0.10 mmol, 59 mg) and AuCl•SMe_2_ (0.11 mmol, 32 mg) in CH_2_Cl_2_ (2 mL) was stirred at room temperature for 24 h under open-air conditions. After filtration of the reaction mixture, a gray solid (36 mg) and filtrate (orange solution) were obtained. The filtrate (orange solution) was evaporated to dryness in vacuo to afford 47 mg of orange solid. On the other hand, 36 mg of gray solid was added to CH_2_Cl_2_ (2 mL), and then the mixture was stirred at room temperature for 24 h. After stirring, the mixture was filtered with suction to give 22 mg of gray solid and filtrate (pale yellow solution). The filtrate was evaporated to dryness in vacuo to afford 10 mg of pale yellow solid. Then, 47 mg of the resulting orange solid and 10 mg of the resulting pale yellow solid were combined. The combined solid (57 mg) thus obtained was purified using column chromatography on silica gel (CH_2_Cl_2_/CH_3_OH = 95/5) to afford 50 mg of white solid. Finally, the recrystallization of the resulting white solid (50 mg) with CH_2_Cl_2_ (2 mL) was performed to afford the desired NHC•AuCl complex **11b** (40 mg, 70% yield).

^1^H NMR (CDCl_3_): δ 7.68 (d, *J* = 8.2 Hz,1H), 7.40–7.25 (m, 9H), 6.34 (br, 1H), 5.68 (s, 2H), 4.78 (t, *J* = 6.6 Hz, 2H), 3.71 (br, 1H), 3.52 (dd, *J* = 5.3 and 11.2 Hz, 1H), 3.45 (dd, *J* = 5.3 and 11.2 Hz, 1H), 3.01 (t, *J* = 6.6 Hz, 2H), 1.47–1.25 (m, 2H), 0.69 (t, *J* = 7.6 Hz, 3H). ^13^C NMR (CDCl_3_): δ 178.2, 169.9, 134.4, 133.4, 132.5, 128.9, 128.5, 127.2, 124.8, 124.7, 112.2, 111.8, 64.4, 53.4, 52.9, 44.9, 37.6, 23.7, 10.2. Anal. Calc. for C_21_H_25_AuClN_3_O_2_: C, 43.20; H, 4.32; N, 7.20. Found: C, 43.47; H, 4.48 N, 7.07%. M.p. 162.0–163.0 °C.

## 4. Conclusions

The switching of enantioselectivity of the catalytic reaction was successfully achieved by changing the order of the addition of the substrates. The effect of the substituents on the chiral ligand and catalytic activity for the reaction of several cyclic enones with dialkylzincs using NHC•HI were comparable to those using NHC•AgI. The present method offers an important advantage of avoiding preparation of the NHC•AgI complex. In addition, the reactions can be performed on benchtop (Schlenck-ware and glove box are not required). Treatment of NHC•AgI with AuCl•SMe_2_ afforded the corresponding NHC•AuCl complex through a ligand transfer reaction. Thus, it can be concluded that the hydroxyamide-functionalized azolium salt, NHC•HX, could be converted into the corresponding monodentate NHC•MX complex (M = Group 11 elements such as Ag and Au), but not NHC•CuX complex

## Figures and Tables

**Table 1:** Initial study.

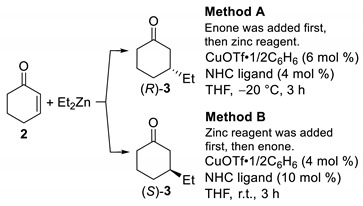			
Entry	NHC Ligand	Method A ^1^	Method B ^2^
entry 1 ^3^	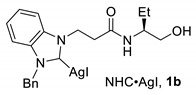	(*R*)-**3** 84% yield 74% ee	(*S*)-**3** 84% yield 87% ee
entry 2	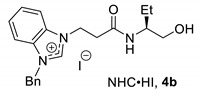	(*R*)-**3** 91% yield 60% ee	(S)-**3** 91% yield 80% ee

^1^ Et_2_Zn (3 mmol) in hexanes (3 mL) was added dropwise to a solution of CuOTf•1/2C_6_H_6_ (0.06 mmol), NHC ligand (0.04 mmol), and **2** (1 mmol) in THF (9 mL) at −20 °C. Then, the reaction mixture was stirred at −20 °C for three hours under Ar. ^2^ **2** (1 mmol) in THF (1.5 mL) was added to a solution of CuOTf•1/2C_6_H_6_ (0.04 mmol), NHC ligand (0.10 mmol), and Et_2_Zn (3 mmol) in THF/hexanes (5.5/3 mL) at 0 °C. Then, the reaction mixture was stirred at room temperature for three hours under air. ^3^ Previously reported data (see [15]).

**Table 2:** Evaluation of various azolium salts.

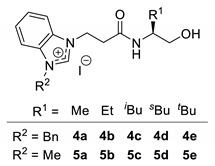					
Entry	Azolium Salt (R^1^)	Method A ^1^		Method B ^2^	
		Product: (*R*)-3		Product: (*S*)-3	
		Yield [%]	Ee [%]	Yield [%]	Ee [%]
entry 1	**4a** (Me)	72	63	77	55
entry 2	**4b** (Et)	91	60	91	80
entry 3	**4c** (*^i^*Bu)	57	65	85	87
entry 4	**4d** (*^s^*Bu)	88	71	78	50
entry 5	**4e** (*^t^*Bu)	90	75	46	−42 ^3^
entry 6	**5a** (Me)	89	50	77	74
entry 7	**5b** (Et)	86	53	80	89
entry 8	**5c** (*^i^*Bu)	88	56	89	86
entry 9	**5d** (*^s^*Bu)	83	60	77	56
entry 10	**5e** (*^t^*Bu)	82	61	80	−36 ^3^

^1^ See Table 1, footnote 1. ^2^ See Table 1, footnote 2. ^3^ (*R*)-**3** was obtained as the major product.

**Table 3:** Evaluation of various cyclic enones using **4b**.

Entry	Method A ^1^	Method B ^2^
entry 1	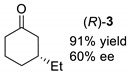	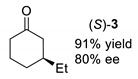
entry 2 ^3^	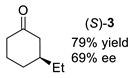	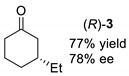
entry 3 ^4^	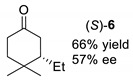	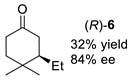
entry 4	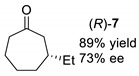	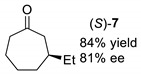
entry 5 ^5^	n.d. ^6^	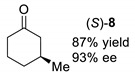
entry 6 ^5^	n.d. ^6^	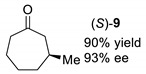

^1^ See Table 1, footnote 1. ^2^ See Table 1, footnote 2. ^3^ *Ent*-**4b** in place of **4b** was used. ^4^ Reaction was conducted for 24 h. ^5^ Me_2_Zn in place of Et_2_Zn was used. ^6^ Almost no reaction was observed.

## Data Availability

The data presented in this study are available in supplementary material.

## Supplementary Materials

The following are available online: Figure S1: ^1^H NMR spectra of (a) product **Y**, (b) NHC∙AgI (**1b**), and (c) NHC∙HI (**4b**) in DMSO-*d*_6_; Spectral data for azolium compounds; NMR charts; Selected chiral GC traces in the catalytic reaction.

## Sample Availability

All products could be prepeared according to the experimental procedure in this manuscript.

## References

[1] Hayashi M., Matsubara R. (2017). Recent topics on catalytic asymmetric 1,4-addition. Tetrahedron Lett..

[2] Alexakis A., Krause N., Woodward S. (2008). Copper-Catalyzed Asymmetric Synthesis.

[3] Jerphagnon T., Pizzuti M.G., Minnaard A.J., Feringa B.L. (2009). Recent advances in enantioselective copper-catalyzed 1,4-addition. Chem. Soc. Rev..

[4] Alexakis A., Bäckvall J.E., Krause N., Pàmies O., Diéguez M. (2008). Enantioselective copper-catalyzed conjugate addition and allylic substitution reactions. Chem. Rev..

[5] Yang G., Zhang W. (2018). Renaissance of pyridine-oxazolines as chiral ligands for asymmetric catalysis. Chem. Soc. Rev..

[6] Janssen-Müller D., Schlepphorst C., Glorius F. (2017). Privileged chiral N-heterocyclic carbene ligands for asymmetric transition-metal catalysis. Chem. Soc. Rev..

[7] Zhou Q.-L. (2011). Privileged Chiral Ligands and Catalysts.

[8] Yoon T.P., Jacobsen E.N. (2003). Privileged chiral catalysis. Science.

[9] Beletskaya I.P., Nájera C., Yus M. (2018). Stereodivergent catalysis. Chem. Rev..

[10] Blanco V., Leigh D.A., Marcos V. (2015). Artificial switchable catalysts. Chem. Soc. Rev..

[11] Escorihuela J., Isabel Burguete M., Luis S.V. (2013). New advances in dual stereocontrol for asymmetric reactions. Chem. Soc. Rev..

[12] Bartók M. (2010). Unexpected inversions in asymmetric reactions: Reactions with chiral metal complexes. Chem. Rev..

[13] Tanaka T., Hayashi M. (2008). Approach for complete reversal of enantioselectivity using a single chiral source. Synthesis.

[14] Zanoni G., Castronovo F., Franzini M., Vidari G., Giannini E. (2003). Toggling enantioselective catalysis-a promising paradigm in the development of more efficient and versatile enantioselective synthetic methodologies. Chem. Soc. Rev..

[15] Nakano Y., Sakaguchi S. (2017). Inversions in asymmetric conjugate addition reaction of cyclic enones catalyzed by the Cu/NHC-AgX system: Factors affecting the stereoselective formation of both enantiomers. J. Organomet. Chem..

[16] Matsumoto K., Nakano Y., Shibata N., Sakaguchi S. (2016). Enantioselectivity switch in copper-catalyzed conjugate addition reactions under the influence of a chiral N-heterocyclic carbene-silver complex. RSC Adv..

[17] Lin J.C.Y., Huang R.T.W., Lee C.S., Bhattacharyya A., Hwang W.S., Lin I.J.B. (2009). Coinage metal-N-heterocyclic carbene complexes. Chem. Rev..

[18] Hahn F.E., Jahnke M.C. (2008). Heterocyclic carbenes: Synthesis and coordination chemistry. Angew. Chem. Int. Ed..

[19] Lin I.J.B., Vasam C.S. (2007). Preparation and application of N-heterocyclic carbene complexes of Ag(I). Coord. Chem. Rev..

[20] De Frémont P., Scott N.M., Stevens E.D., Ramnial T., Lightbody O.C., Macdonald C.L.B., Clyburne J.A.C., Abernethy C.D., Nolan S.P. (2005). Synthesis of well-defined N-heterocyclic carbene silver(I) complexes. Organometallics.

[21] Garrison J.C., Youngs W.J. (2005). Ag(I) N-heterocyclic carbene complexes: Synthesis, structure, and application. Chem. Rev..

[22] Budagumpi S., Keri R.S., Achar G., Brinda K.N. (2020). Coinage metal complexes of chiral N-heterocyclic carbene ligands: Syntheses and applications in asymmetric catalysis. Adv. Synth. Catal..

[23] Khose V.N., John M.E., Pandey A.D., Karnik A.V. (2017). Chiral benzimidazoles and their applications in stereodiscrimination processes. Tetrahedron Asymmetry.

[24] Wang F., Liu L.-j., Wang W., Li S., Shi M. (2012). Chiral NHC–metal-based asymmetric catalysis. Coord. Chem. Rev..

[25] Gade L.H., Bellemin-Laponnaz S. (2007). Mixed oxazoline-carbenes as stereodirecting ligands for asymmetric catalysis. Coord. Chem. Rev..

[26] César V., Bellemin-Laponnaz S., Gade L.H. (2004). Chiral N-heterocyclic carbenes as stereodirecting ligands in asymmetric catalysis. Chem. Soc. Rev..

[27] Perry M.C., Burgess K. (2003). Chiral N-heterocyclic carbene-transition metal complexes in asymmetric catalysis. Tetrahedron Asymmetry.

[28] Herrmann W.A. (2002). N-Heterocyclic carbenes: A new concept in organometallic catalysis. Angew. Chem. Int. Ed..

[29] Magrez M., Wencel-Delord J., Alexakis A., Crévisy C., Mauduit M. (2012). Significant asymmetric amplification in enantioselective Cu/DiPPAM-catalyzed 1,6- and 1,4-conjugate additions of diethylzinc to (di)enone. Org. Lett..

[30] Satyanarayana T., Abraham S., Kagan H.B. (2009). Nonlinear effects in asymmetric catalysis. Angew. Chem. Int. Ed..

[31] Luukas T.O., Fenwick D.R., Kagan H.B. (2002). Presence or absence of a nonlinear effect according to the asymmetric catalyst preparation in the alkylation of benzaldehyde. C. R. Chim..

[32] Walsh P.J. (2003). Titanium-Catalyzed Enantioselective Additions of Alkyl Groups to Aldehydes: Mechanistic Studies and New Concepts in Asymmetric Catalysis. Acc. Chem. Res..

[33] Li D., Ollevier T. (2020). Mechanism studies of oxidation and hydrolysis of Cu(I)-NHC and Ag-NHC in solution under air. J. Organomet. Chem..

[34] Li D., Ollevier T. (2019). Synthesis of imidazolidinone, imidazolone, and benzimidazolone derivatives through oxidation using copper and air. Org. Lett..

[35] Cervantes-Reyes A., Rominger F., Hashmi A.S.K. (2020). Sterically demanding Ag(I) and Cu(I) N-heterocyclic carbine complexes: Synthesis, structures, steric parameters and catalytic activity. Chem. Eur. J..

[36] Johnson A., Gimeno M.C. (2016). An efficient and sustainable synthesis of NHC gold complexes. Chem. Commun..

[37] Marion N., Nolan S.P. (2008). N-Heterocyclic carbenes in gold catalysis. Chem. Soc. Rev..

[38] Shibata N., Yoshimura M., Yamada H., Arakawa R., Sakaguchi S. (2012). Hydroxy-amide functionalized azolium salts for Cu-catalyzed asymmetric conjugate addition: Stereocontrol based on ligand structure and copper precatalyst. J. Org. Chem..

