# Supplementary material for: Reversal of Enantioselectivity in the Conjugate Addition Reaction of Cyclic Enones with the CuOTf/Azolium Catalytic System
Source: Molecules. 2021 Jun 4;26(11):3404. doi: 10.3390/molecules26113404 (PMC8200092; doi:10.3390/molecules26113404)
Supplement: Supplementary file 1 [file molecules-26-03404-s001.zip › molecules-1239374-supplementary.pdf]

molecules

MDPI

Supplementary Materials

Reversal of Enantioselectivity in the Conjugate Addition Reaction of Cyclic Enones with the CuOTf/Azolium Catalytic System

Yuki Nakano, Satoki Shimizu, Chihiro Takeda and Satoshi Sakaguchi*

Department of Chemistry and Materials Engineering, Faculty of Chemistry, Materials and Bioengineering, Kansai University, Suita, Osaka 564-8680, Japan; k729143@kansai-u.ac.jp (Y.N.); k323441@kansai-u.ac.jp (S.S.); k422297@kansai-u.ac.jp (C.T.)

* Correspondence: satoshi@kansai-u.ac.jp

(a) Compound **Y** [(NHC•H⁺)(CuX₂⁻)]

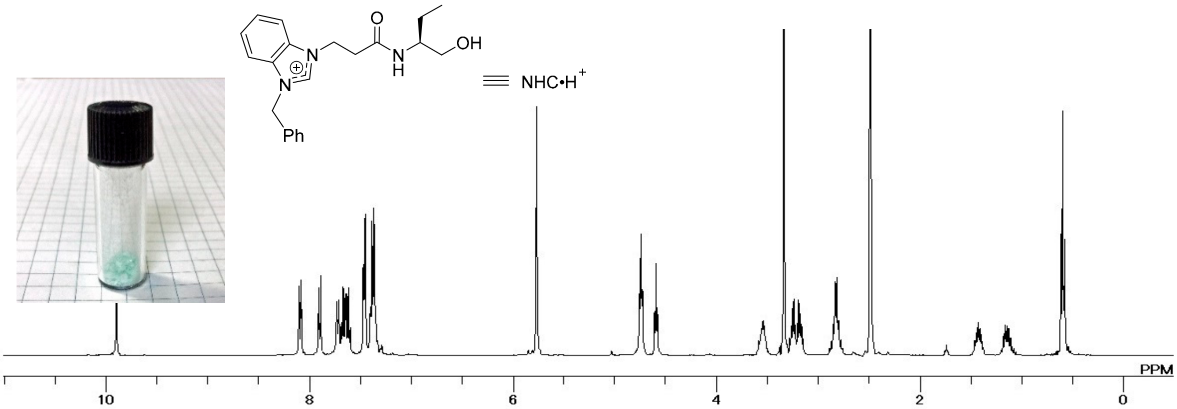

(b) NHC·AgI (**1b**)

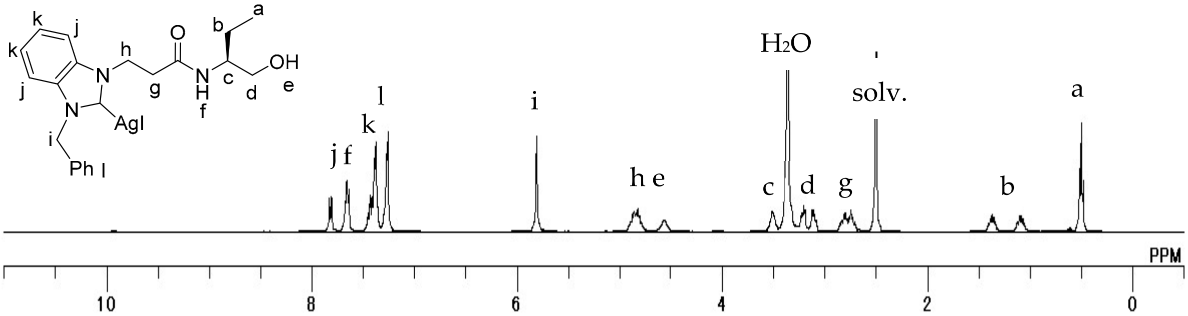

(c) NHC·HI (**4b**)

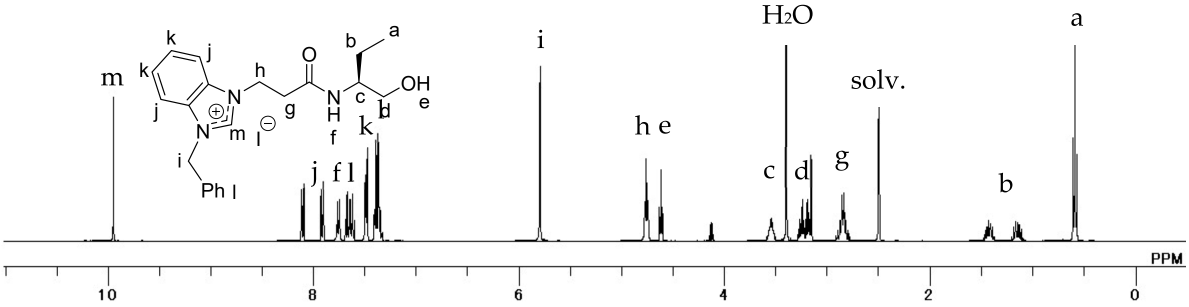

Figure S1. ^{1}H NMR spectra of (**a**) compound **Y**, (**b**) NHC·AgI (**1b**) and (**c**) NHC·HI (**4b**) in DMSO-d_6.

2. Spectral data for azolium compounds

4a (R^1=Me, R^2=Bn)

^{1}H-NMR ($CDCl_3$, 400 MHz): δ 10.48 (s, 1H), 7.88 (d, *J* = 8.1 Hz, 1H), 7.63-7.48 (m, 6H), 7.40-7.31 (m, 3H), 5.80 (d, *J* = 15.4 Hz, 1H), 5.71 (d, *J* = 15.4 Hz, 1H), 4.97-4.92 (m, 1H), 4.86-4.82 (m, 1H), 3.93-3.86 (m, 1H), 3.61-3.54 (m, 1H), 3.49-3.40 (m, 3H), 3.11-3.06 (m, 2H), 1.05 (d, *J* = 6.3 Hz, 3H); ^{13}C-NMR ($CDCl_3$, 100 MHz): δ 141.7, 132.1, 131.8, 130.9, 129.4, 128.3, 127.3, 113.6, 113.3, 65.3, 51.8, 48.2, 44.1, 35.4, 16.5.

4b (R^1=Et, R^2=Bn)

^{1}H-NMR ($CDCl_3$, 400 MHz): δ 10.51 (s, 1H), 7.88 (d, *J* = 8.2 Hz, 1H), 7.62-7.57 (m, 1H), 7.52-7.44 (m, 5H), 7.40-7.33 (m, 3H), 5.80 (d, *J* = 15.1 Hz, 1H), 5.74 (d, *J* = 15.1 Hz, 1H), 4.99-4.93 (m, 1H), 4.88-4.81 (m, 1H), 3.77-3.69 (m, 1H), 3.51-3.44 (m, 3H), 3.31 (br, 1H), 3.17-3.09 (m, 1H), 1.47-1.34 (m, 2H), 0.75 (t, *J* = 7.3 Hz, 3H); ^{13}C-NMR ($CDCl_3$, 100 MHz): δ 169.2, 141.7, 132.1, 131.8, 130.9, 129.4, 129.4, 128.3, 127.4, 127.3, 113.6, 113.3, 63.7, 53.9, 51.8, 44.0, 35.3, 23.8, 10.5.

Ent-**4b** (R^1=Et, R^2=Bn)

^{1}H-NMR ($CDCl_3$, 400 MHz): δ 10.52 (s, 1H), 7.89 (d, *J* = 8.7 Hz, 1H), 7.64-7.57 (m, 1H), 7.54-7.43 (m, 5H), 7.40-7.33 (m, 3H), 5.80 (d, *J* = 15.1 Hz, 1H), 5.75 (d, *J* = 15.1 Hz, 1H), 5.00-4.93 (m, 1H), 4.88-4.82 (m, 1H), 3.77-3.69 (m, 1H), 3.52-3.42 (m, 3H), 3.29 (br, 1H), 3.16-3.10 (m, 1H), 1.47-1.36 (m, 2H), 0.75 (t, *J* = 7.5 Hz, 3H); ^{13}C-NMR ($CDCl_3$, 100 MHz): δ 169.4, 141.9, 132.3, 131.8, 130.9, 129.4, 129.3, 128.3, 127.3, 127.2, 113.7, 113.4, 63.7, 53.8, 51.9, 44.1, 35.5, 23.8, 10.5.

4c (R^1=^{i}Bu, R^2=Bn)

^{1}H-NMR ($CDCl_3$, 400 MHz): δ 10.45 (s, 1H), 7.91 (d, *J* = 8.2 Hz, 1H), 7.59-7.56 (m, 1H), 7.50-7.47 (m, 4H),7.38-7.32 (m, 4H), 5.78 (d, *J* = 15.1 Hz, 1H), 5.73 (d, *J* = 15.1 Hz, 1H), 4.97-4.92 (m, 1H), 4.86-4.81 (m, 1H), 3.94-3.91 (m, 1H), 3.50-3.40 (m, 3H), 3.26-3.25 (br, 1H), 3.12-3.07 (m, 1H), 1.43-1.30 (m, 2H), 1.16-1.10 (m, 1H), 0.78 (d, *J* = 6.2 Hz, 3H), 0.74 (d, *J* = 6.2 Hz, 3H); ^{13}C-NMR ($CDCl_3$, 100 MHz): δ 169.2, 142.0, 132.3, 131.8, 130.9, 129.4, 129.3, 128.3, 127.3, 127.2, 113.7, 113.4, 64.6, 51.9, 50.4, 44.1, 39.7, 35.5, 24.7, 22.8, 22.1.

4d (R^1=^{s}Bu, R^2=Bn)

^{1}H-NMR ($CDCl_3$, 400 MHz): δ 10.55 (s, 1H), 7.88 (d, *J* = 8.5 Hz, 1H), 7.61-7.57 (m, 1H), 7.52-7.49 (m, 4H), 7.40-7.34 (m, 4H), 5.80 (d, *J* = 15.0 Hz, 1H), 5.74 (d, *J* = 15.0 Hz, 1H), 5.00-4.95 (m, 1H), 4.88-4.85 (m, 1H), 3.71-3.66 (m, 1H), 3.62-3.51 (m, 2H), 3.45-3.42 (m, 1H), 3.23 (br, 1H), 3.15-3.08 (m, 1H), 1.57-1.55 (m, 1H), 1.35-1.31 (m, 1H), 1.02-0.95 (m, 1H), 0.79-0.74 (m, 6H); ^{13}C-NMR ($CDCl_3$, 100 MHz): δ 169.2, 141.9, 132.2, 131.7, 130.8, 129.3, 129.2, 128.2, 127.2, 127.1, 113.6, 113.3, 61.8, 56.3, 51.8, 44.1, 35.4, 35.4, 25.6, 15.2, 11.1.

4e (R^1=^{t}Bu, R^2=Bn)

^{1}H-NMR ($CDCl_3$, 400 MHz): δ 10.54 (s, 1H), 7.86 (d, *J* = 8.1 Hz, 1H), 7.63-7.59 (m, 1H), 7.54-7.47 (m, 4H), 7.40-7.36 (m, 3H), 7.21 (d, *J* = 9.4 Hz, 1H), 5.80 (d, *J* = 15.3 Hz, 1H), 5.70 (d, *J* = 15.3 Hz, 1H), 5.04-4.97 (m, 1H), 4.79-4.76 (m, 1H), 3.82-3.67 (m, 3H), 3.60-3.56 (m, 1H), 3.11-3.04 (m, 2H), 0.86 (s, 9H); ^{13}C-NMR ($CDCl_3$, 100 MHz): δ 169.7, 141.8, 132.2, 131.8, 130.8, 129.3, 129.3, 128.2, 127.3, 127.2, 113.6, 113.3, 60.6, 60.1, 51.8, 43.9, 35.1, 33.5, 26.9.

5a (R^1=Me, R^2=Me)

^{1}H-NMR (DMSO, 400 MHz): δ 9.66 (s, 1H), 8.09-8.05 (m, 1H), 8.03-8.00 (m, 1H), 7.81 (d, *J* = 8.1 Hz, 1H), 7.82-7.66 (m, 2H), 4.71-4.68 (m, 2H), 4.63 (t, *J* = 5.5 Hz, 1H) 4.07 (s, 3H), 3.75-3.65 (m, 1H), 3.24-3.19 (m, 1H), 3.16-3.12 (m, 1H), 2.75 (t, *J* = 7.3 Hz, 2H), 0.89 (d, *J* = 7.3 Hz, 3H); ^{13}C-NMR (DMSO, 100 MHz): δ 168.3, 143.1 131.6, 130.8, 126.4, 126.4, 113.6, 113.5, 64.1, 46.5, 43.2, 34.3, 33.2, 16.9.

5b (R^1=Et, R^2=Me)

^{1}H-NMR (DMSO, 400 MHz): δ 9.67 (s, 1H), 8.10-8.07 (m, 1H), 8.02-7.99 (m, 1H), 7.73-7.68 (m, 3H), 4.72 (t, *J* = 6.4 Hz, 2H), 4.58 (t, *J* = 5.5 Hz, 1H), 4.08 (s, 3H), 3.59-3.51 (m, 1H), 3.26-3.15 (m, 2H), 2.86-2.74 (m, 2H), 1.49-1.38 (m, 1H), 1.22-1.10 (m, 1H), 0.64 (t, *J* = 7.3 Hz, 3H); ^{13}C-NMR (DMSO, 100 MHz): δ 168.7, 143.1, 131.6, 130.7, 126.5, 126.4, 113.6, 113.5, 62.6, 52.2, 43.3, 34.4, 33.2, 23.4, 10.1.

5c (R^1=^{i}Bu, R^2=Me)

^{1}H-NMR (DMSO, 400 MHz): δ 9.65 (s, 1H), 8.08-8.05 (m, 1H), 8.03-7.99 (m, 1H), 7.71-7.68 (m, 3H), 4.76-4.66 (m, 2H), 4.58 (br, 1H), 4.07 (s, 3H), 3.74-3.69 (m, 1H), 3.49-3.34 (m, 2H), 2.86-2.72 (m, 2H), 1.22-1.04 (m, 3H), 0.71 (d, *J* = 7.3 Hz, 3H), 0.67 (d, *J* = 7.3 Hz, 3H); ^{13}C-NMR (DMSO, 100 MHz): δ 168.3, 143.1, 131.6, 130.7, 126.5, 126.4, 113.6, 113.4, 63.6, 48.7, 43.4, 39.7, 34.4, 33.2, 24.0, 23.1, 21.6.

5d (R^1=^{s}Bu, R^2=Me)

^{1}H-NMR (CDCl$_3$, 400 MHz): δ 10.29 (s, 1H), 7.95-7.89 (m, 1H), 7.74-7.68 (m, 1H), 7.68-7.60 (m, 2H), 7.33 (d, *J* = 8.5 Hz, 1H), 5.00-4.93 (m, 1H), 4.89-4.83 (m, 1H), 4.20 (s, 3H), 3.72-3.66 (m, 1H), 3.62-3.55 (m, 1H), 3.48-3.41 (m, 2H), 3.31 (t, *J* = 5.6 Hz, 1H), 3.16-3.09 (m, 1H), 1.58-1.48 (m, 1H), 1.36-1.26 (m, 1H), 1.06-0.93 (m, 1H), 0.78-0.74 (m, 6H); ^{13}C-NMR (CDCl$_3$, 100 MHz): δ 169.4, 142.1, 131.4, 131.1, 127.0, 126.9, 113.5, 112.5, 61.5, 55.9, 43.8, 35.5, 35.2, 34.2, 25.4, 15.0, 11.0.

5e (R^1=^{t}Bu, R^2=Me)

^{1}H-NMR (CDCl$_3$, 400 MHz): δ 10.29 (s, 1H), 7.91-7.88 (m, 1H), 7.70-7.63 (m, 3H), 7.18 (d, *J* = 9.6 Hz, 1H), 5.03-4.96 (m, 1H), 4.80-4.74 (m, 1H), 4.19 (s, 3H), 3.82-3.77 (m, 1H), 3.70-3.59 (m, 3H), 3.21 (br, 1H), 3.10-3.04 (m, 1H), 0.87 (s, 9H); ^{13}C-NMR (CDCl$_3$, 100 MHz): δ 169.9, 142.1, 131.7, 131.5, 127.4, 127.3, 113.5, 112.5, 60.6, 60.1, 43.8, 35.3, 34.1, 33.6, 26.9.

10b (R^1=Et, R^2=Bn)

^{1}H-NMR (CDCl$_3$, 400 MHz): δ 10.77 (s, 1H), 7.92-7.86 (m, 2H), 7.60-7.56 (m, 1H), 7.54-7.47 (m, 4H), 7.38-7.32 (m, 3H), 5.82 (d, *J* = 15.3 Hz, 1H), 5.76 (d, *J* = 15.3 Hz, 1H), 4.99-4.92 (m, 1H), 4.88-4.82 (m, 1H), 3.91 (br, 1H), 3.66 (br, 1H), 3.50-3.38 (m, 3H), 3.14-3.07 (m, 1H), 1.43-1.36 (m, 2H), 0.73 (t, *J* = 7.4 Hz, 3H); ^{13}C-NMR (CDCl$_3$, 100 MHz): δ 169.1, 142.3, 132.4, 131.7, 130.7, 129.2, 129.1, 128.1, 127.0, 127.0, 113.5, 113.3, 63.4, 53.6, 51.5, 44.0, 35.3, 23.7, 10.3.

Ent-**10b** (R^1=Et, R^2=Bn)

^{1}H-NMR (CDCl$_3$, 400 MHz): δ 10.77 (s, 1H), 7.90-7.86 (m, 2H), 7.60-7.54 (m, 1H), 7.52-7.47 (m, 4H), 7.37-7.30 (m, 3H), 5.82 (d, *J* = 15.1 Hz, 1H), 5.75 (d, *J* = 15.1 Hz, 1H), 4.98-4.92 (m, 1H), 4.87-4.81 (m, 1H), 3.88 (br, 1H), 3.68-3.62 (m, 1H), 3.50-3.37 (m, 3H), 3.13-3.06 (m, 1H), 1.45-1.34 (m, 2H), 0.74 (t, *J* = 7.4 Hz, 3H); ^{13}C-NMR (CDCl$_3$, 100 MHz): δ 169.1, 142.3, 132.4, 131.7, 130.7, 129.2, 129.1, 128.1, 127.0, 127.0, 113.5, 113.3, 63.4, 53.6, 51.5, 44.0, 35.3, 23.7, 10.3.

3. NMR chart

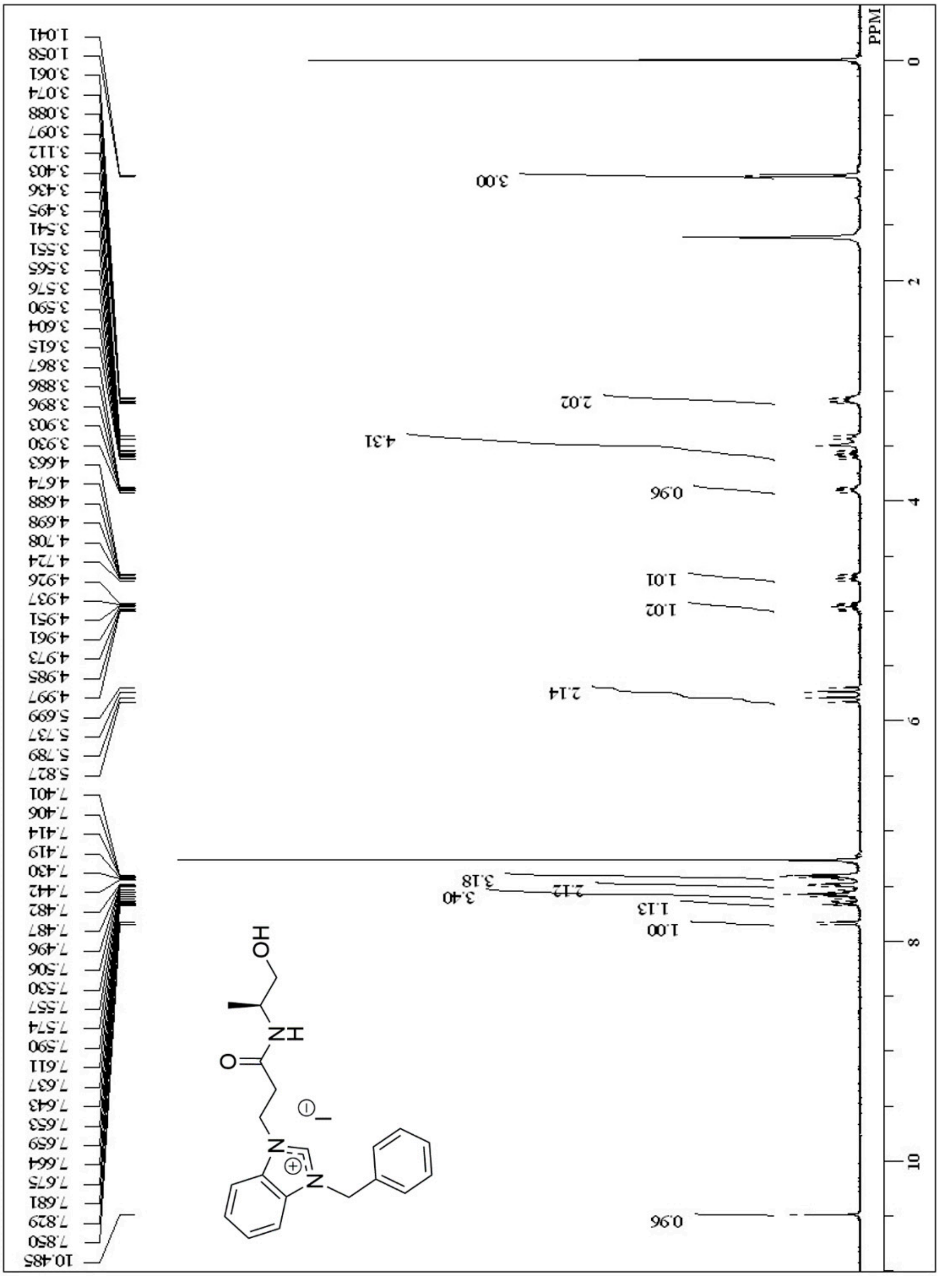
1.041
1.058
3.061
3.074
3.088
3.097
3.112
3.403
3.436
3.495
3.541
3.551
3.565
3.576
3.590
3.604
3.615
3.867
3.886
3.896
3.903
3.930
4.663
4.674
4.688
4.698
4.708
4.724
4.926
4.937
4.951
4.961
4.973
4.985
4.997
5.699
5.737
5.789
5.827
7.401
7.406
7.414
7.419
7.430
7.442
7.482
7.487
7.496
7.506
7.530
7.557
7.574
7.590
7.611
7.637
7.643
7.653
7.659
7.664
7.675
7.681
7.829
7.850
10.485
3.00
2.02
4.31
0.96
1.01
1.02
2.14
3.18
2.12
3.40
1.13
1.00
0.96
PPM
0
2
4
6
8
10
OH
N
H
O
I
N
N

4a (R[1]=Me, R[2]=Bn).

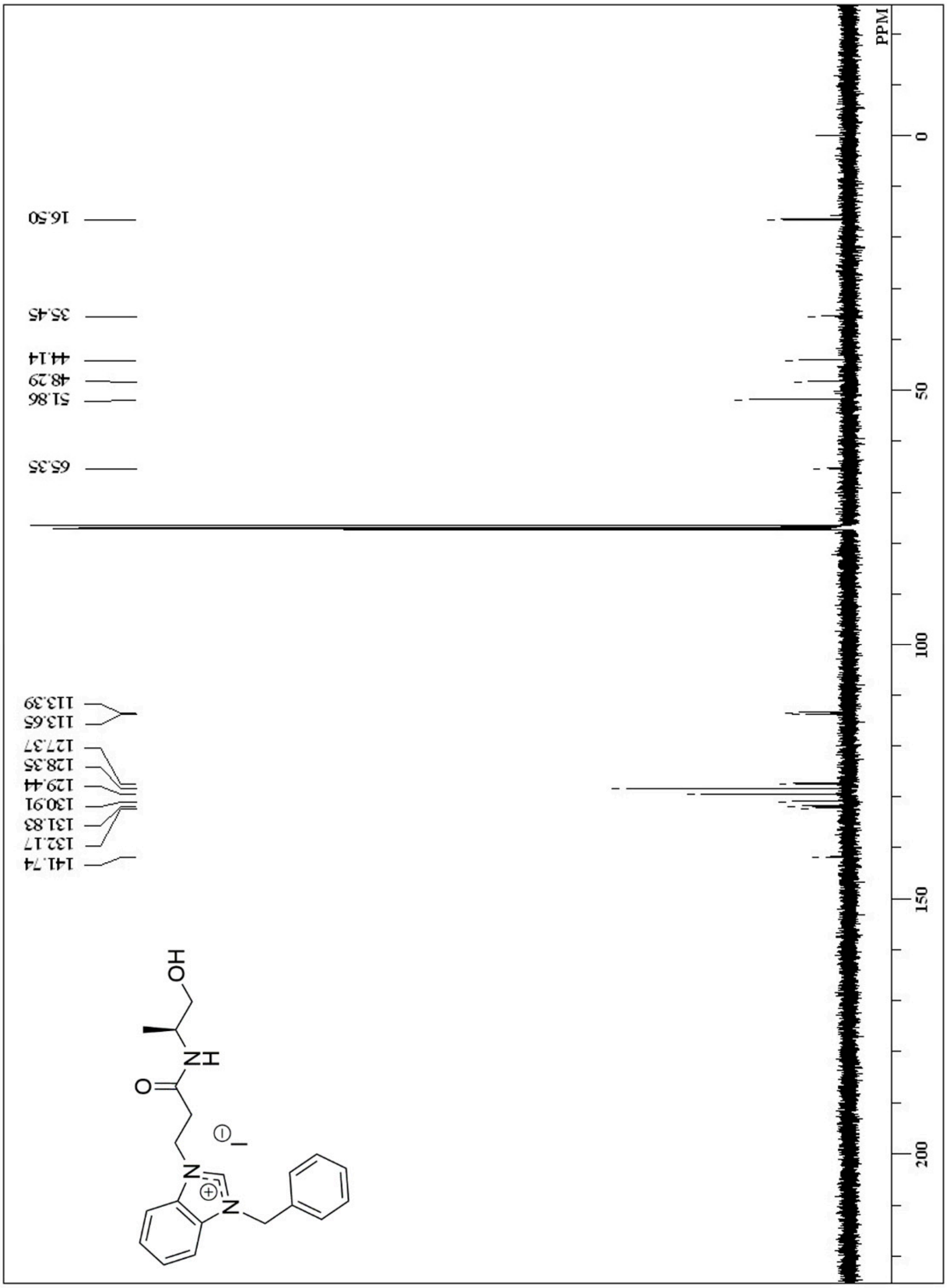
16.50
35.45
44.14
48.29
51.86
65.35
113.39
113.65
127.37
128.35
129.44
130.91
131.83
132.17
141.74
PPM
0
50
100
150
200

4a (R[1]=Me, R[2]=Bn).

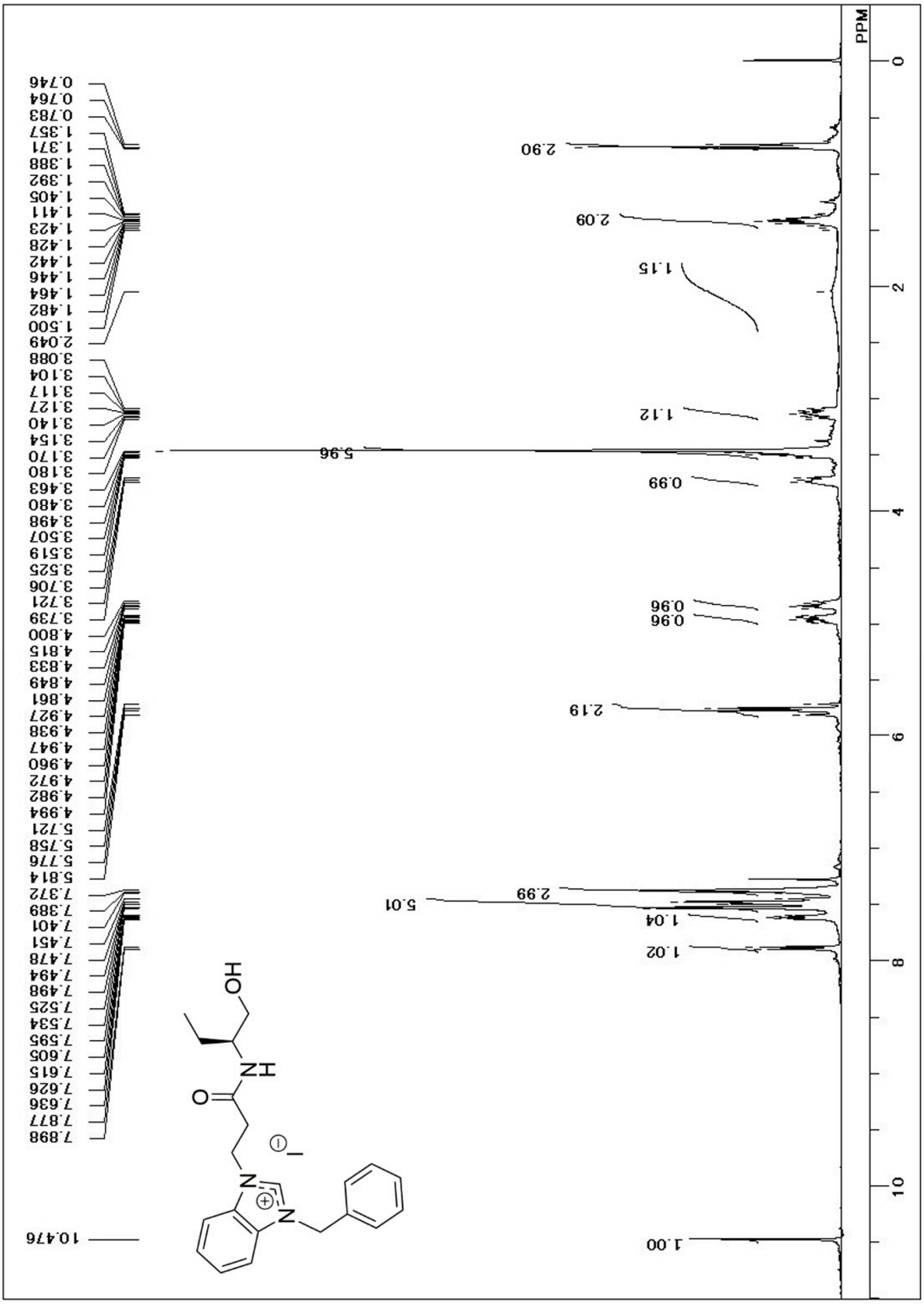
0.746
0.764
0.783
1.357
1.371
1.388
1.392
1.405
1.411
1.423
1.428
1.442
1.446
1.464
1.482
1.500
2.049
3.088
3.104
3.117
3.127
3.140
3.154
3.170
3.180
3.463
3.480
3.498
3.507
3.519
3.525
3.706
3.721
3.739
4.800
4.815
4.833
4.849
4.861
4.927
4.938
4.947
4.960
4.972
4.982
4.994
5.721
5.758
5.776
5.814
7.372
7.389
7.401
7.451
7.478
7.494
7.498
7.525
7.534
7.595
7.605
7.615
7.626
7.636
7.877
7.898
10.476
2.90
2.09
1.15
1.12
5.96
0.99
0.96
0.96
2.19
2.99
5.01
1.04
1.02
1.00
PPM
0
2
4
6
8
10
OH
H
N
O
I
N
N
+

4b (R[1]=Et, R[2]=Bn).

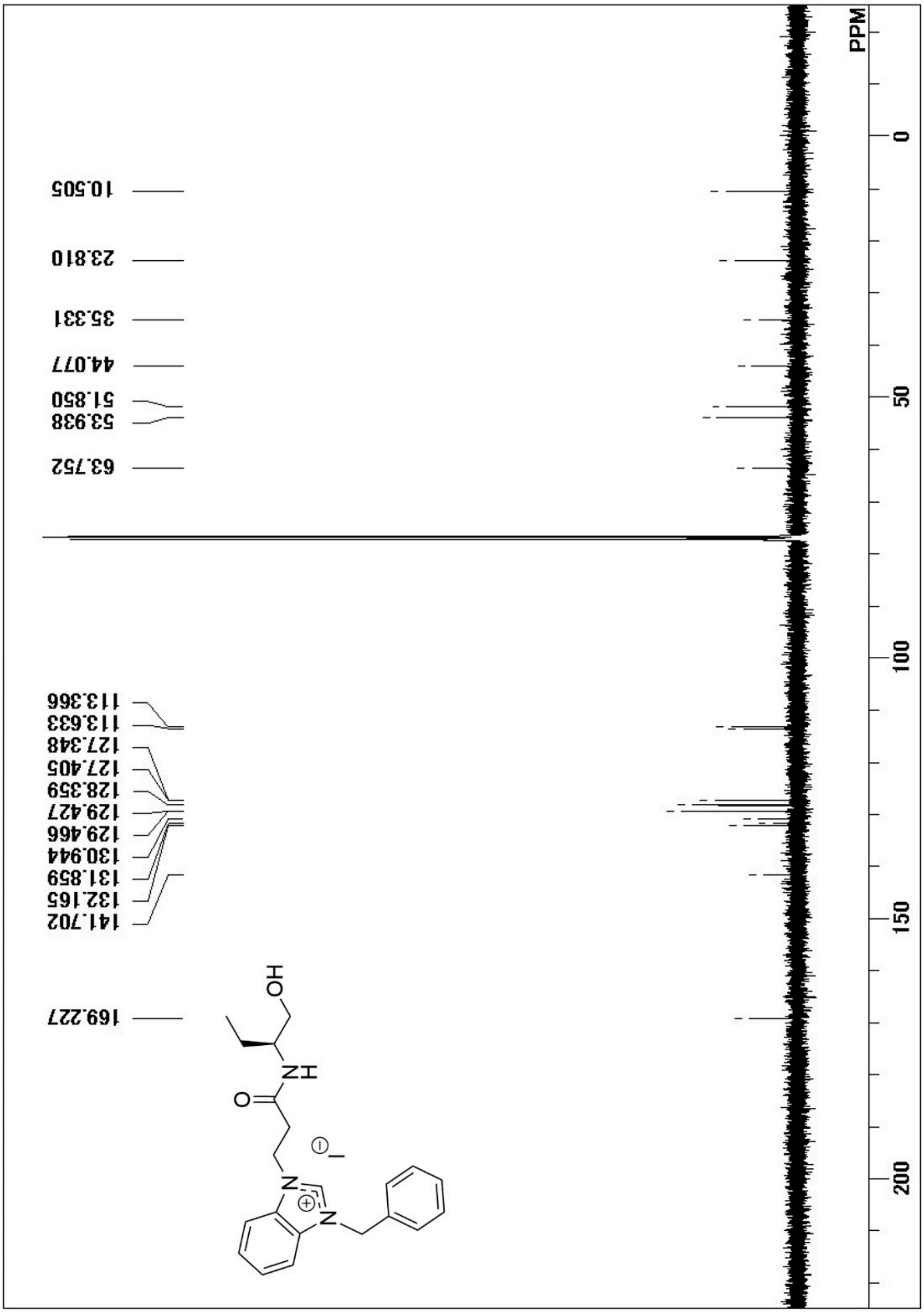
10.505
23.810
35.331
44.077
51.850
53.938
63.752
113.366
113.633
127.348
127.405
128.359
129.427
129.466
130.944
131.859
132.165
141.702
169.227
PPM
0
50
100
150
200

4b (R^1=Et, R^2=Bn).

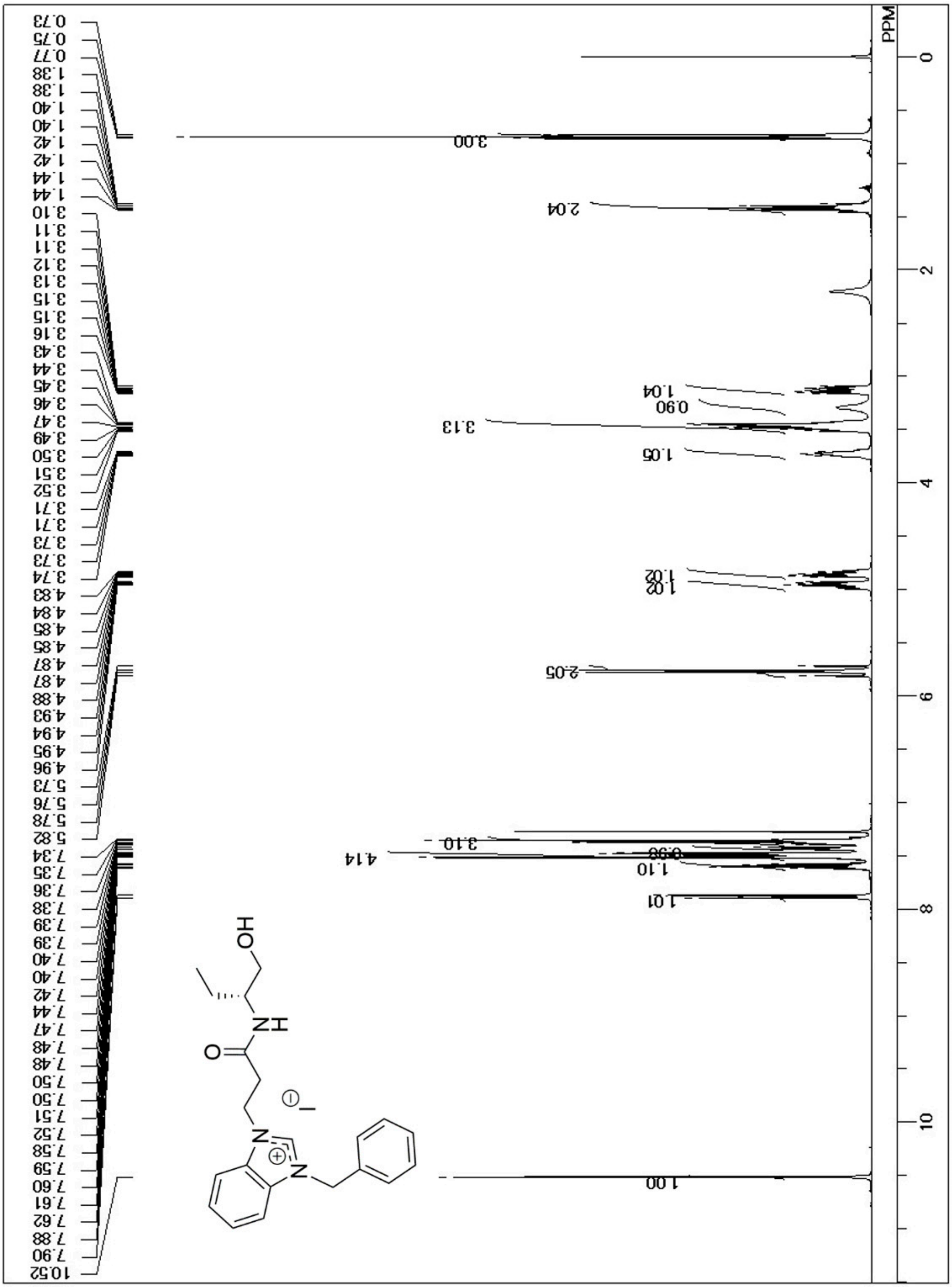

***Ent*-4b (R[1]=Et, R[2]=Bn).**

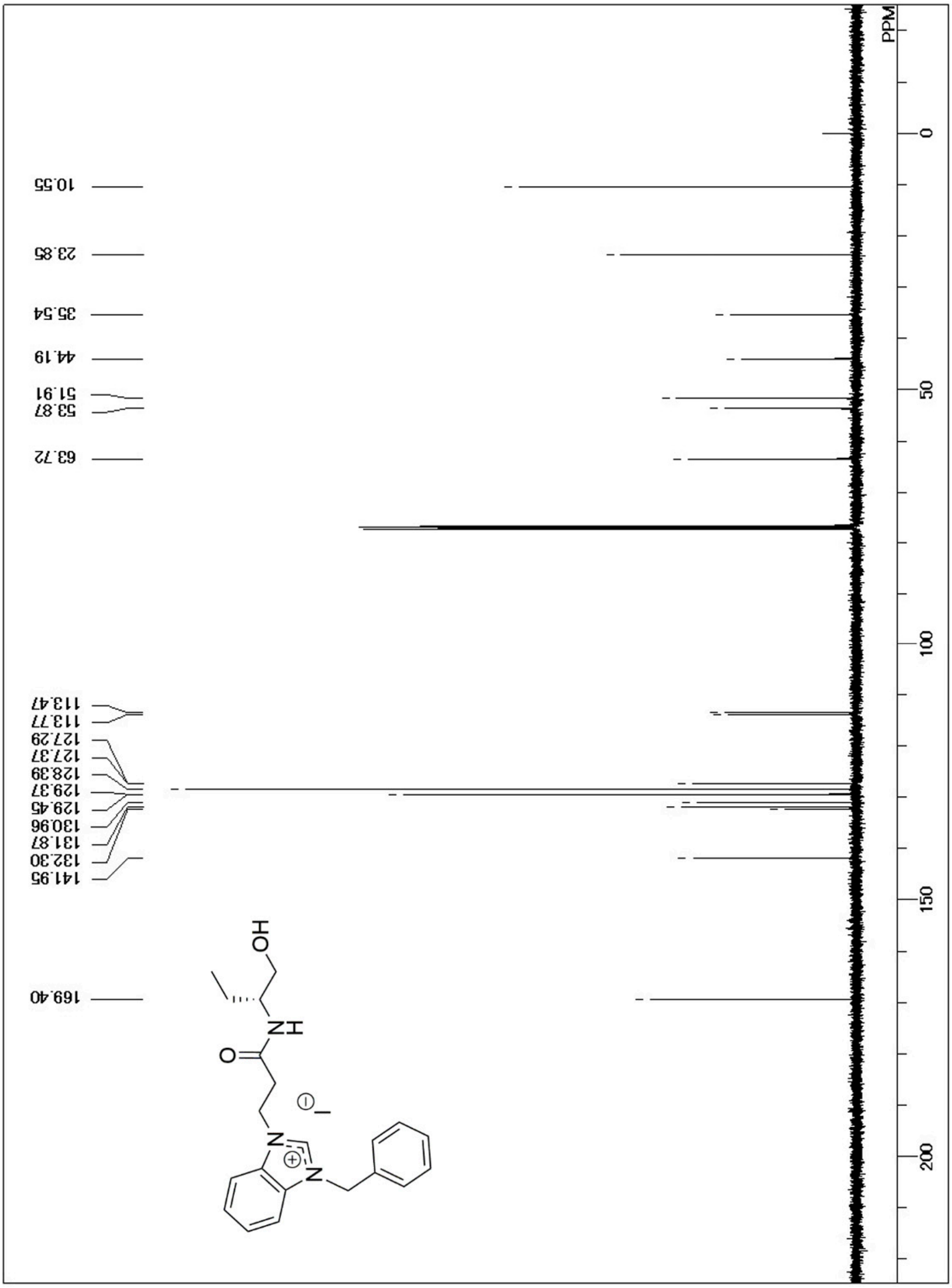

Ent-4b (R[1]=Et, R[2]=Bn).

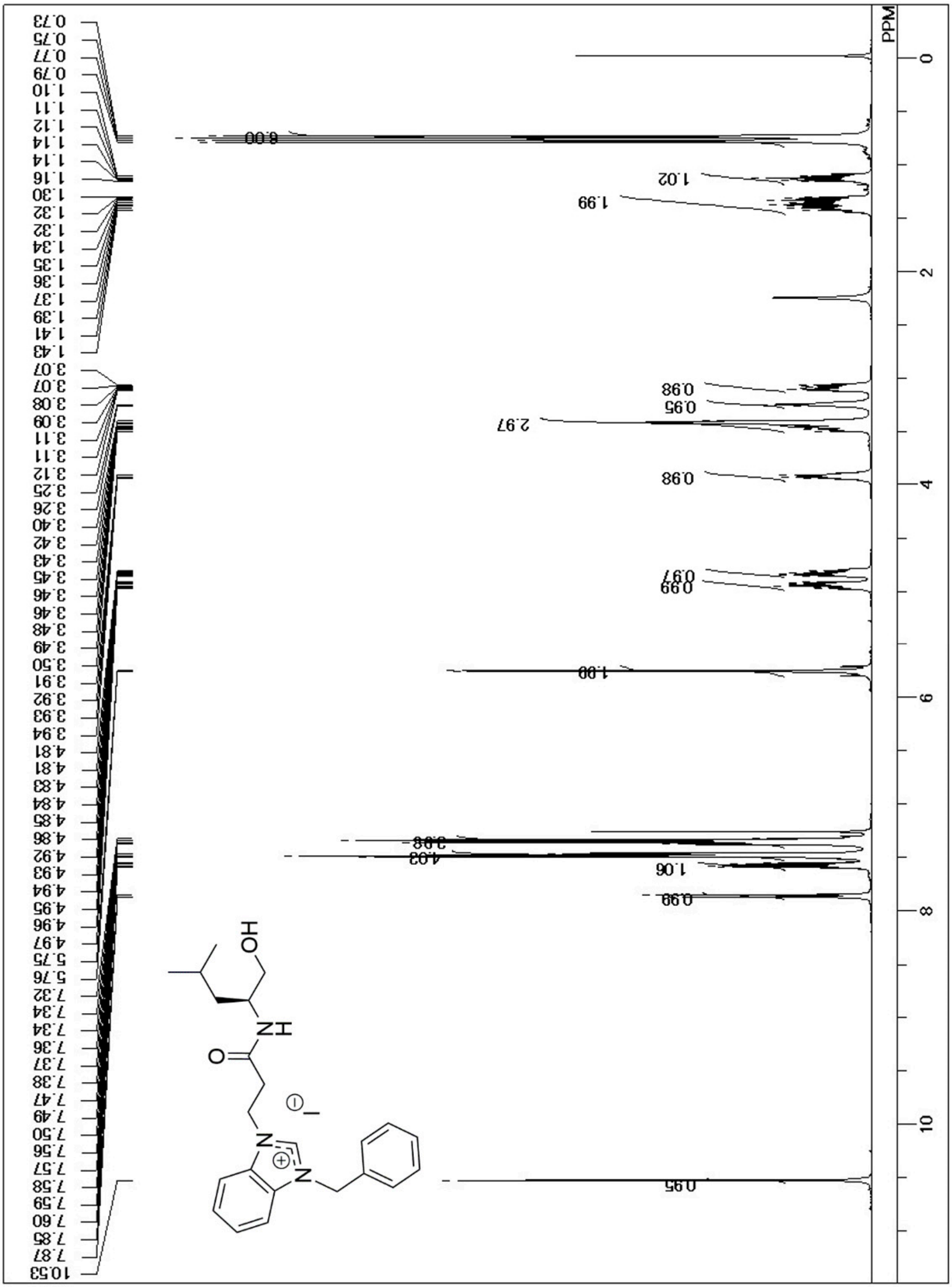

4c (R[1]=^{i}Bu, R[2]=Bn).

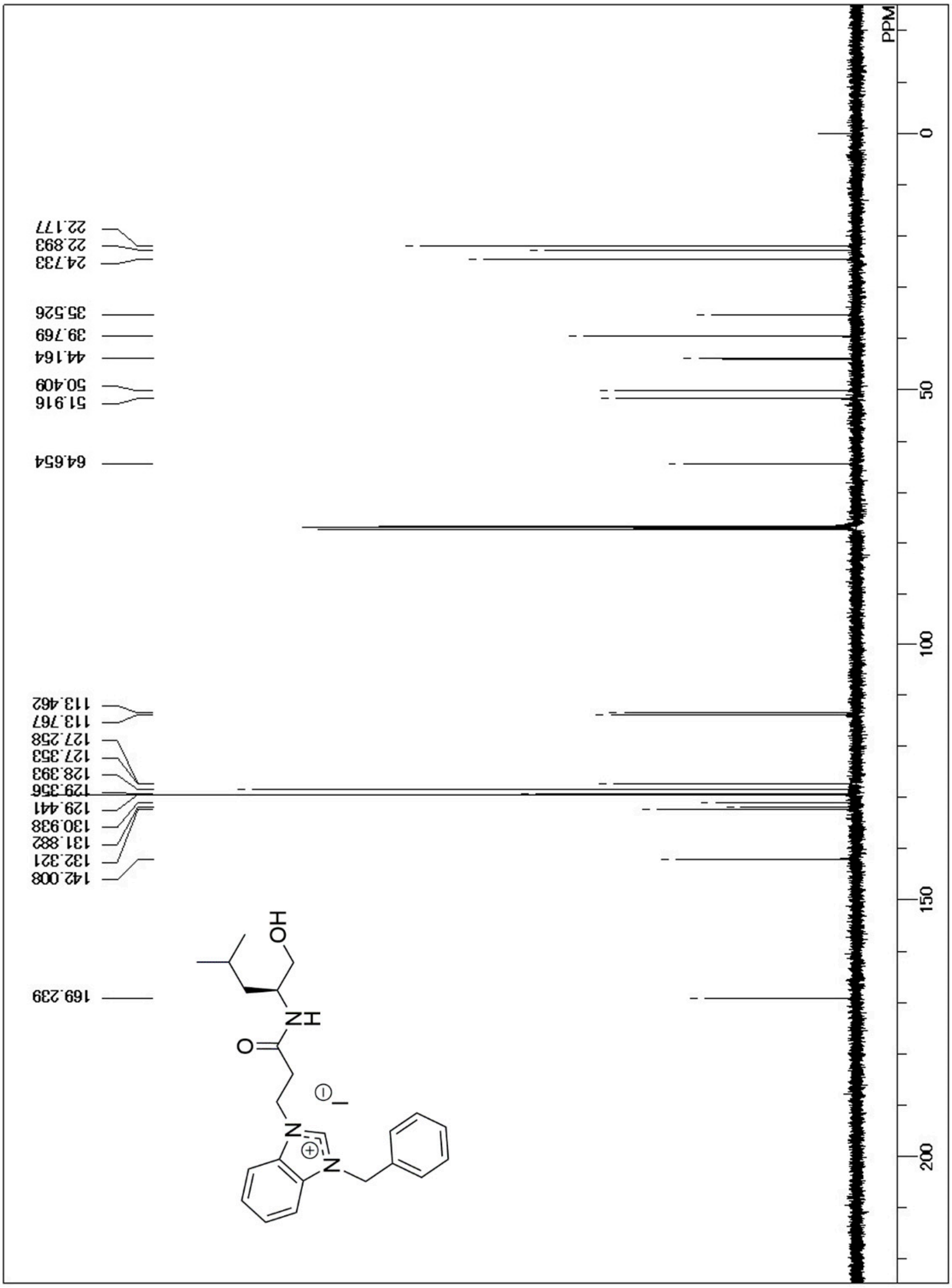

4c (R^1=^{i}Bu, R^2=Bn).

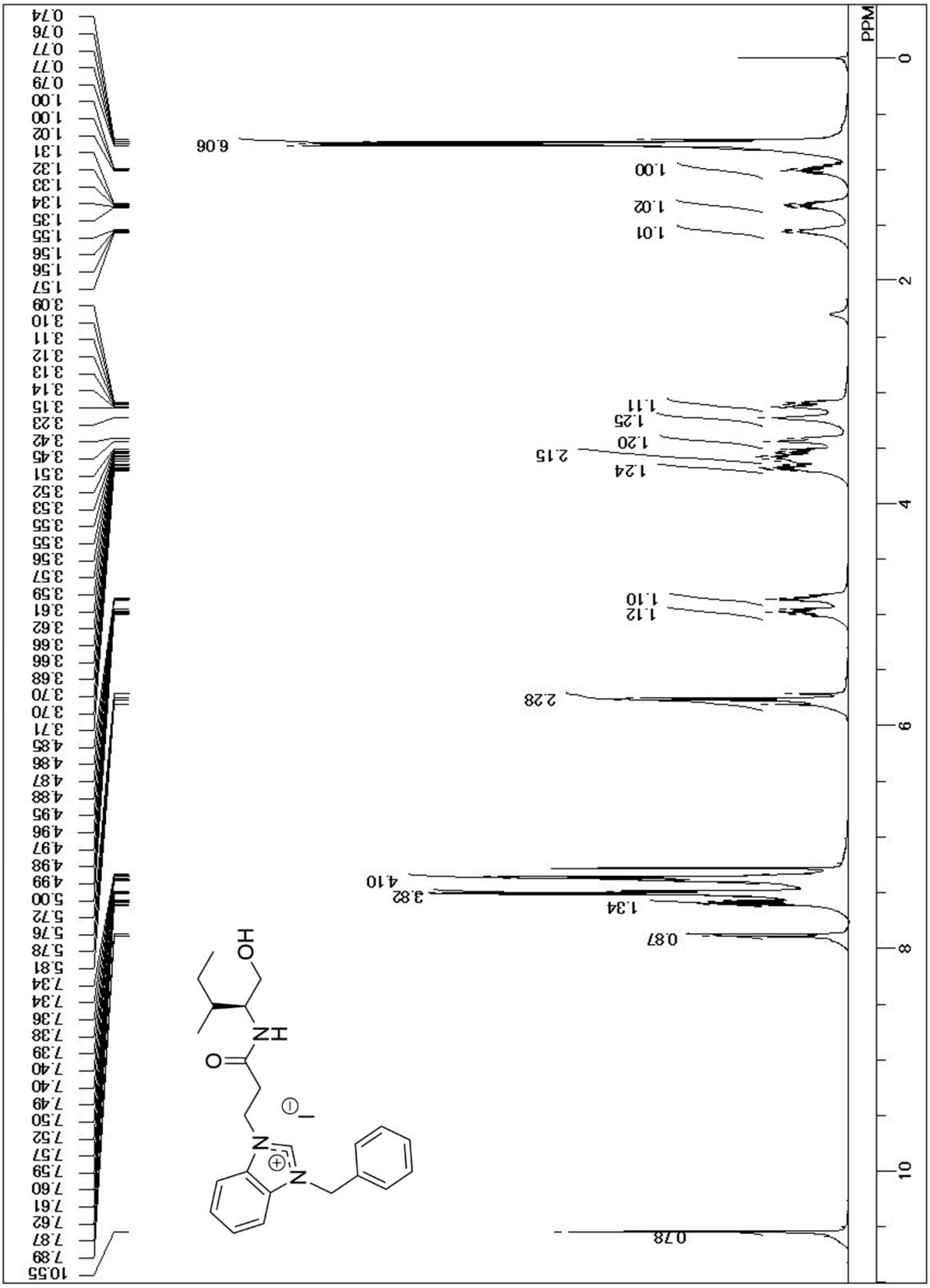

4d (R[1]=[s]Bu, R[2]=Bn).

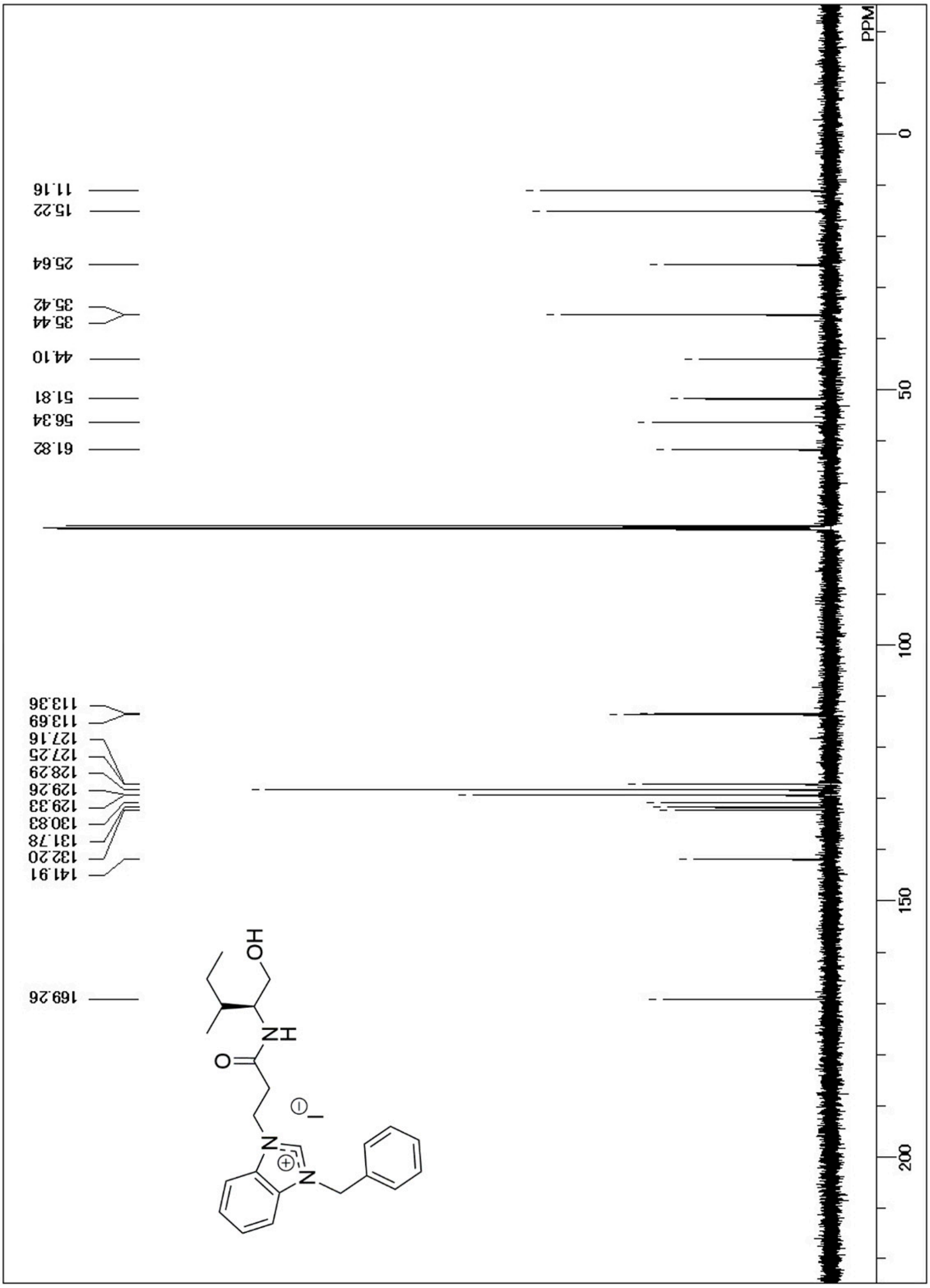

4d (R^1=^{s}Bu, R^2=Bn).

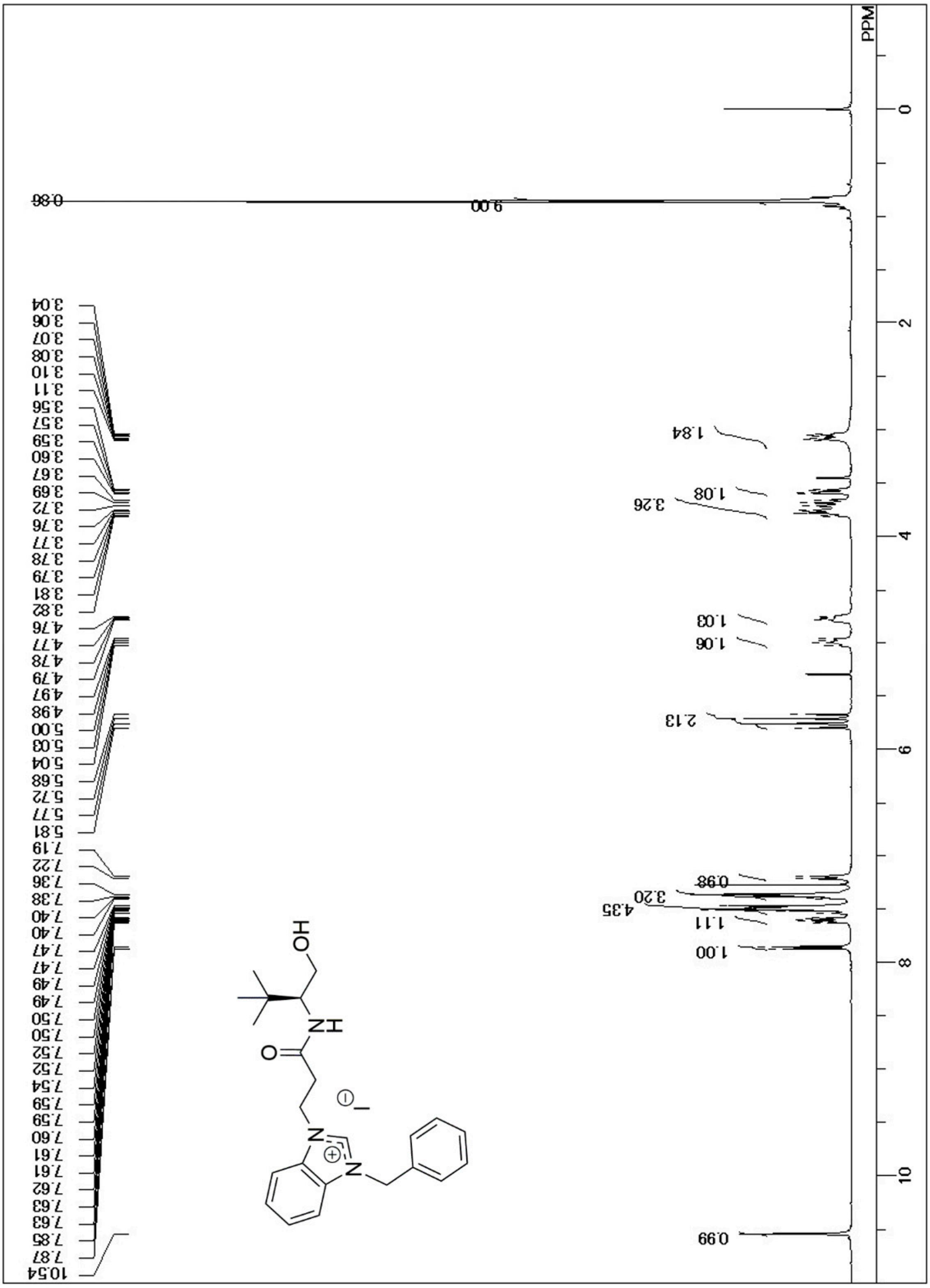

4e (R[1]=^{t}Bu, R[2]=Bn).

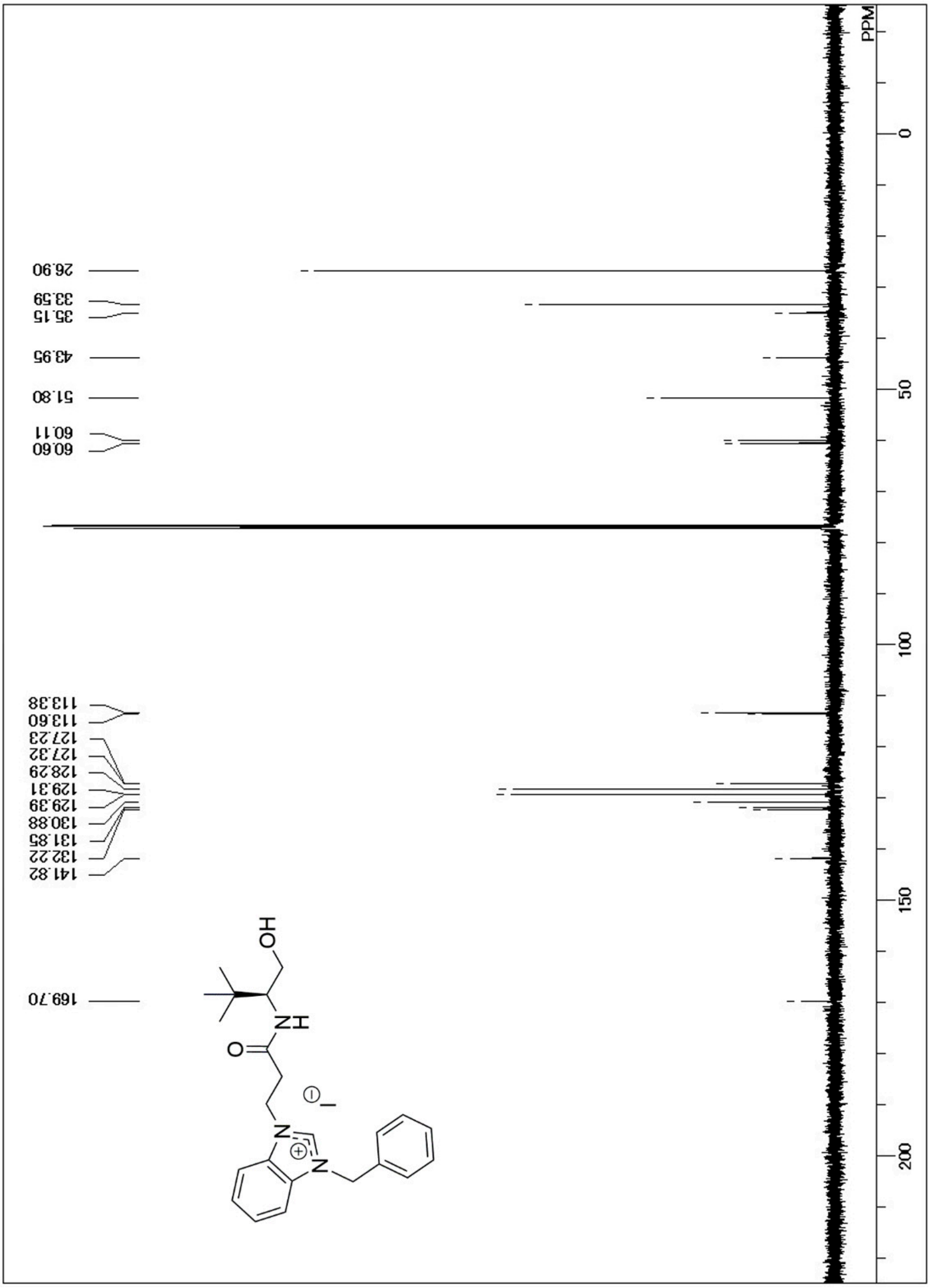

4e (R^1=^{t}Bu, R^2=Bn).

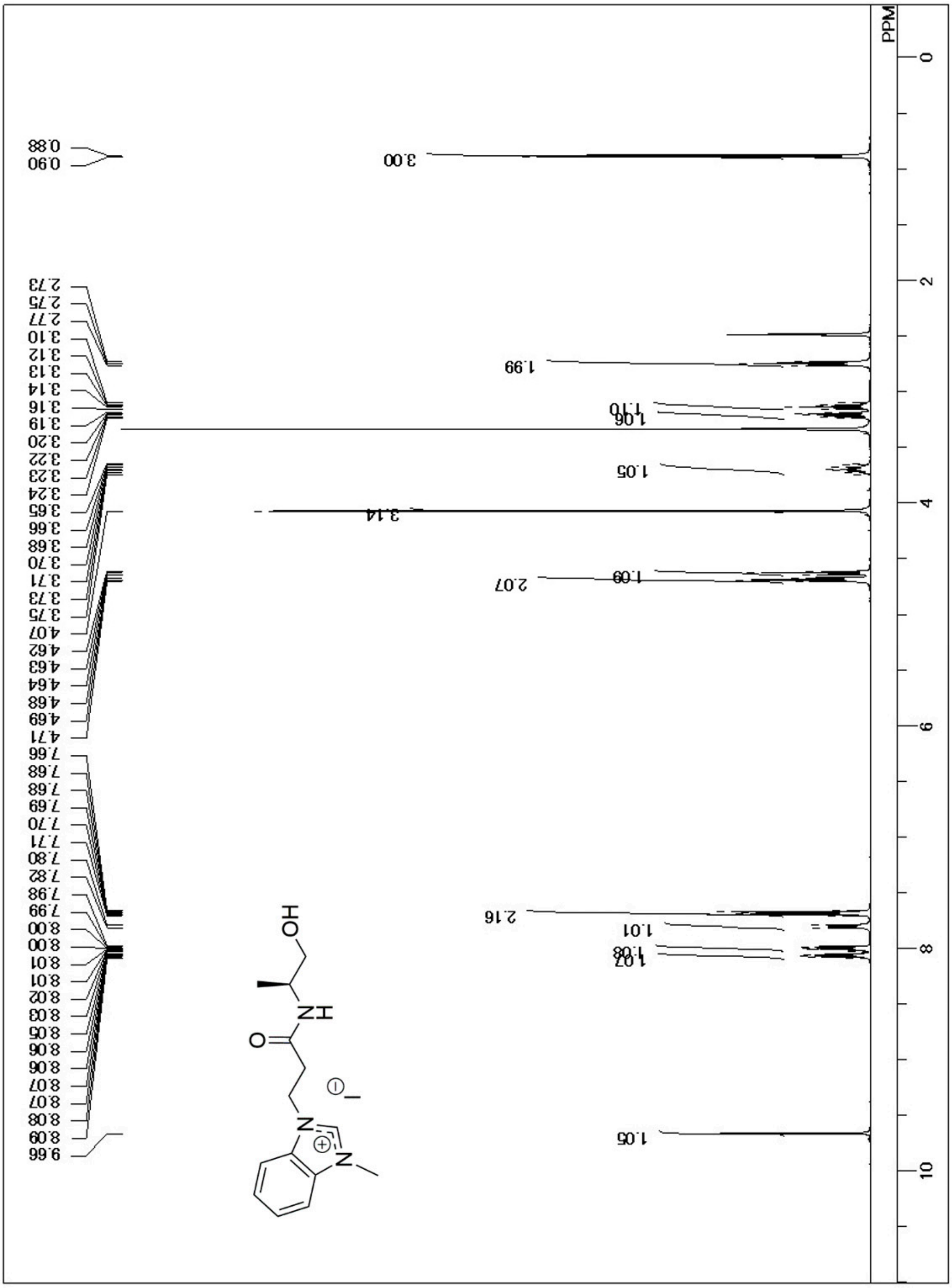
0.88
0.90
2.73
2.75
2.77
3.10
3.12
3.13
3.14
3.16
3.19
3.20
3.22
3.23
3.24
3.65
3.66
3.68
3.70
3.71
3.73
3.75
4.07
4.62
4.63
4.64
4.68
4.69
4.71
7.66
7.68
7.68
7.69
7.70
7.71
7.80
7.82
7.98
7.99
8.00
8.00
8.01
8.01
8.02
8.03
8.05
8.06
8.06
8.07
8.07
8.08
8.09
9.66
3.00
1.99
1.10
1.06
1.05
3.14
2.07
1.09
2.16
1.01
1.08
1.07
1.05
PPM
0
2
4
6
8
10
HO
NH
O
N
N
I

5a (R¹=Me, R²=Me).

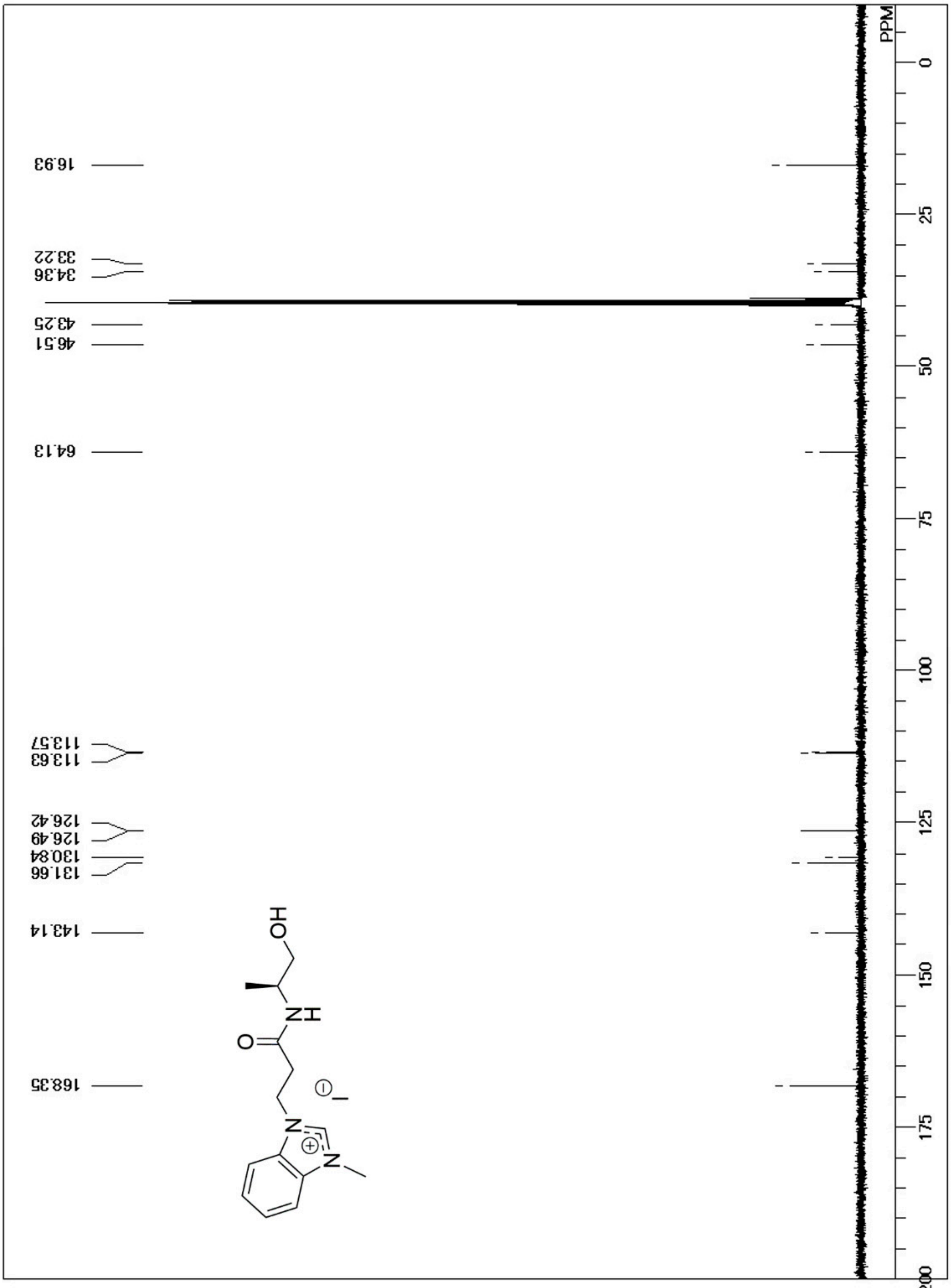
16.93
33.22
34.36
43.25
46.51
64.13
113.57
113.63
126.42
126.49
130.84
131.66
143.14
168.35
PPM
0
25
50
75
100
125
150
175
200

5a (R^1=Me, R^2=Me).

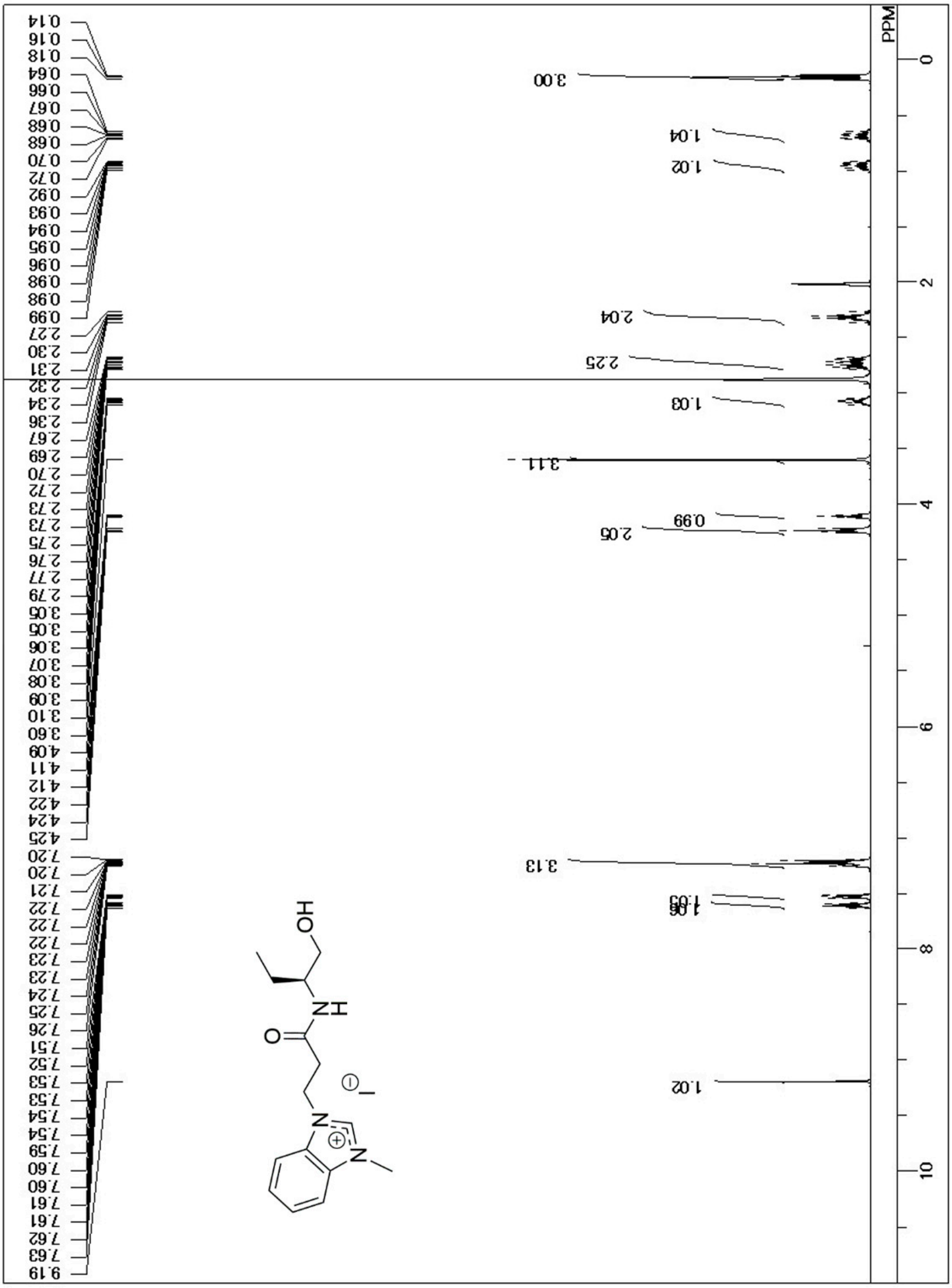
0.14 0.16 0.18 0.64 0.66 0.67 0.68 0.68 0.70 0.72 0.92 0.93 0.94 0.95 0.96 0.98 0.98 0.99 2.27 2.30 2.31 2.32 2.34 2.36 2.67 2.69 2.70 2.72 2.73 2.73 2.75 2.76 2.77 2.79 3.05 3.05 3.06 3.07 3.08 3.09 3.10 3.60 4.09 4.11 4.12 4.22 4.24 4.25 7.20 7.20 7.21 7.22 7.22 7.22 7.23 7.23 7.24 7.25 7.26 7.51 7.52 7.53 7.53 7.54 7.54 7.59 7.60 7.60 7.61 7.61 7.62 7.63 9.19
3.00
1.04
1.02
2.04
2.25
1.03
3.11
0.99
2.05
3.13
1.05
1.06
1.02
PPM
0
2
4
6
8
10
OH
N
H
O
N
N
I

5b (R¹=Et, R²=Me).

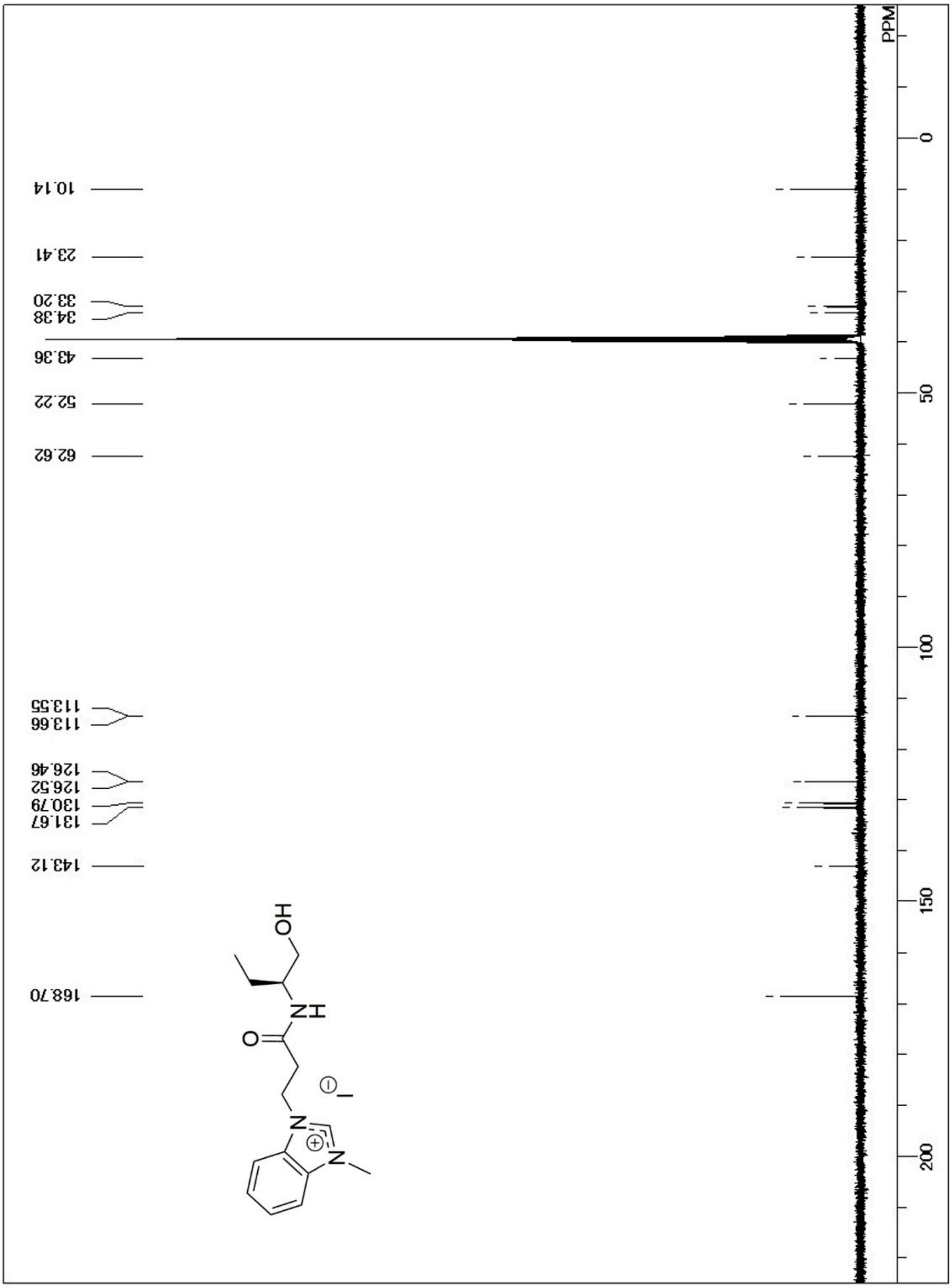
10.14
23.41
33.20
34.38
43.36
52.22
62.62
113.55
113.66
126.46
126.52
130.79
131.67
143.12
168.70
PPM
0
50
100
150
200

5b (R[1]=Et, R[2]=Me).

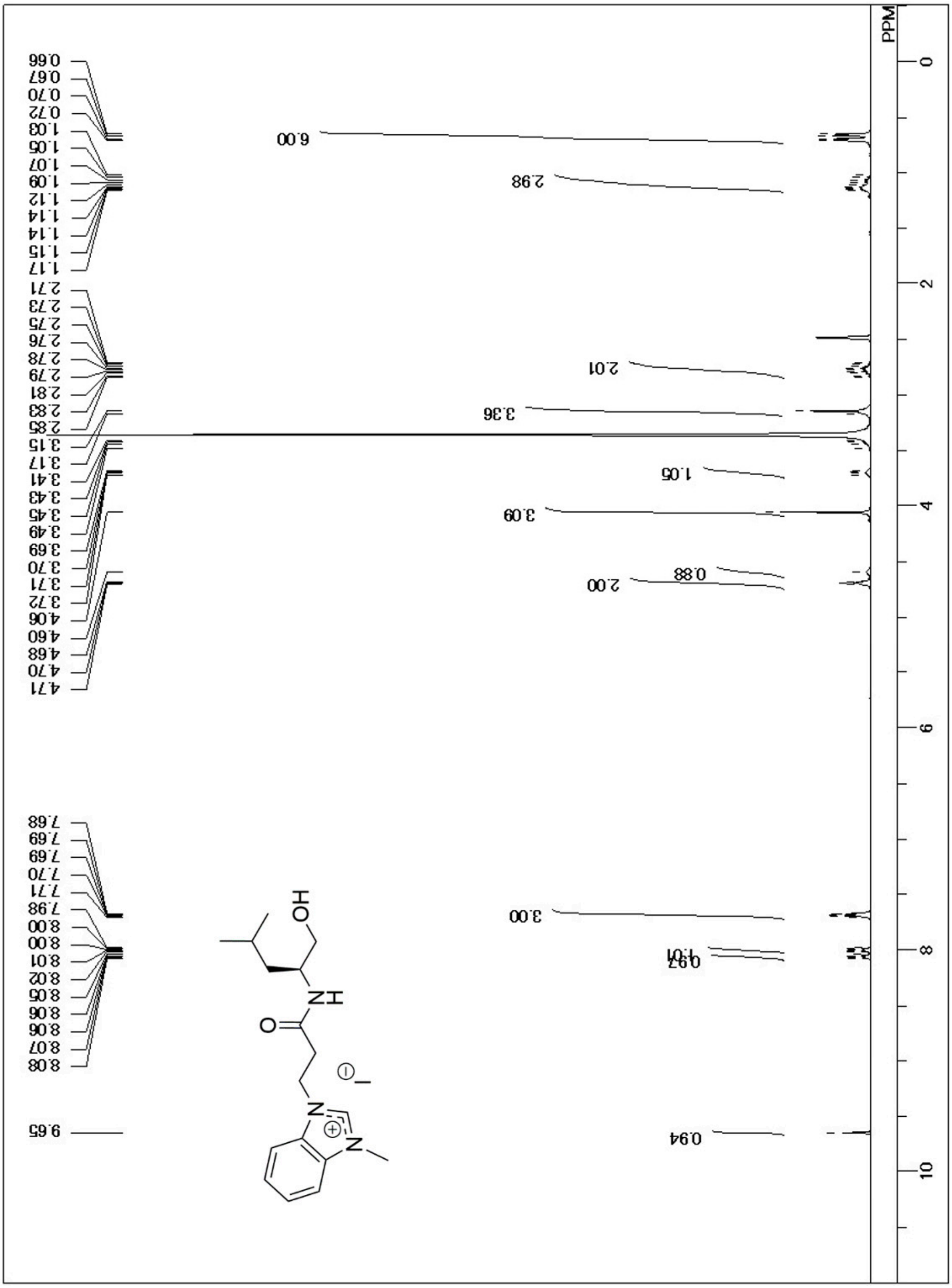

5c (R^1=^{i}Bu, R^2=Me).

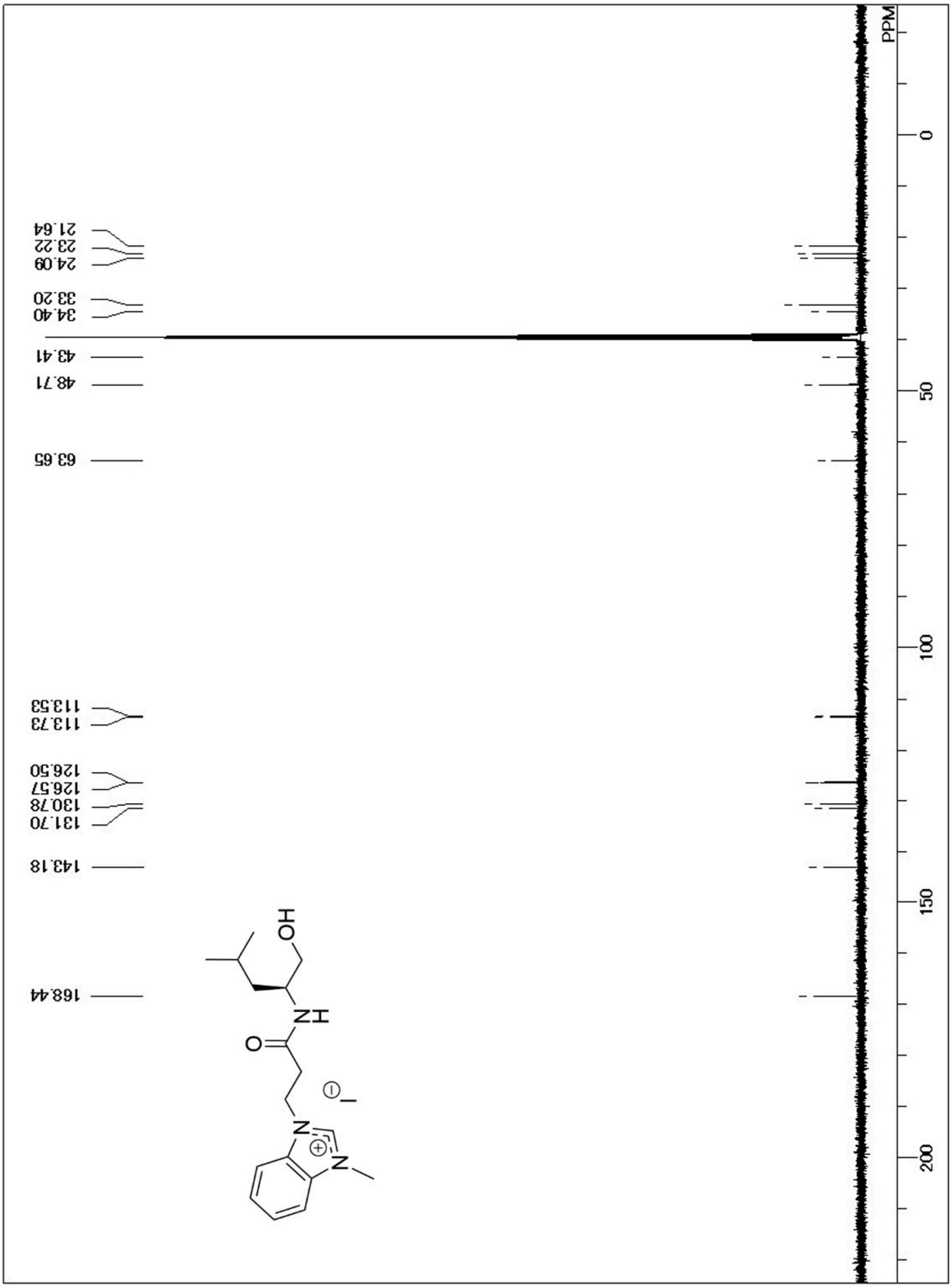

5c (R^1=^{i}Bu, R^2=Me).

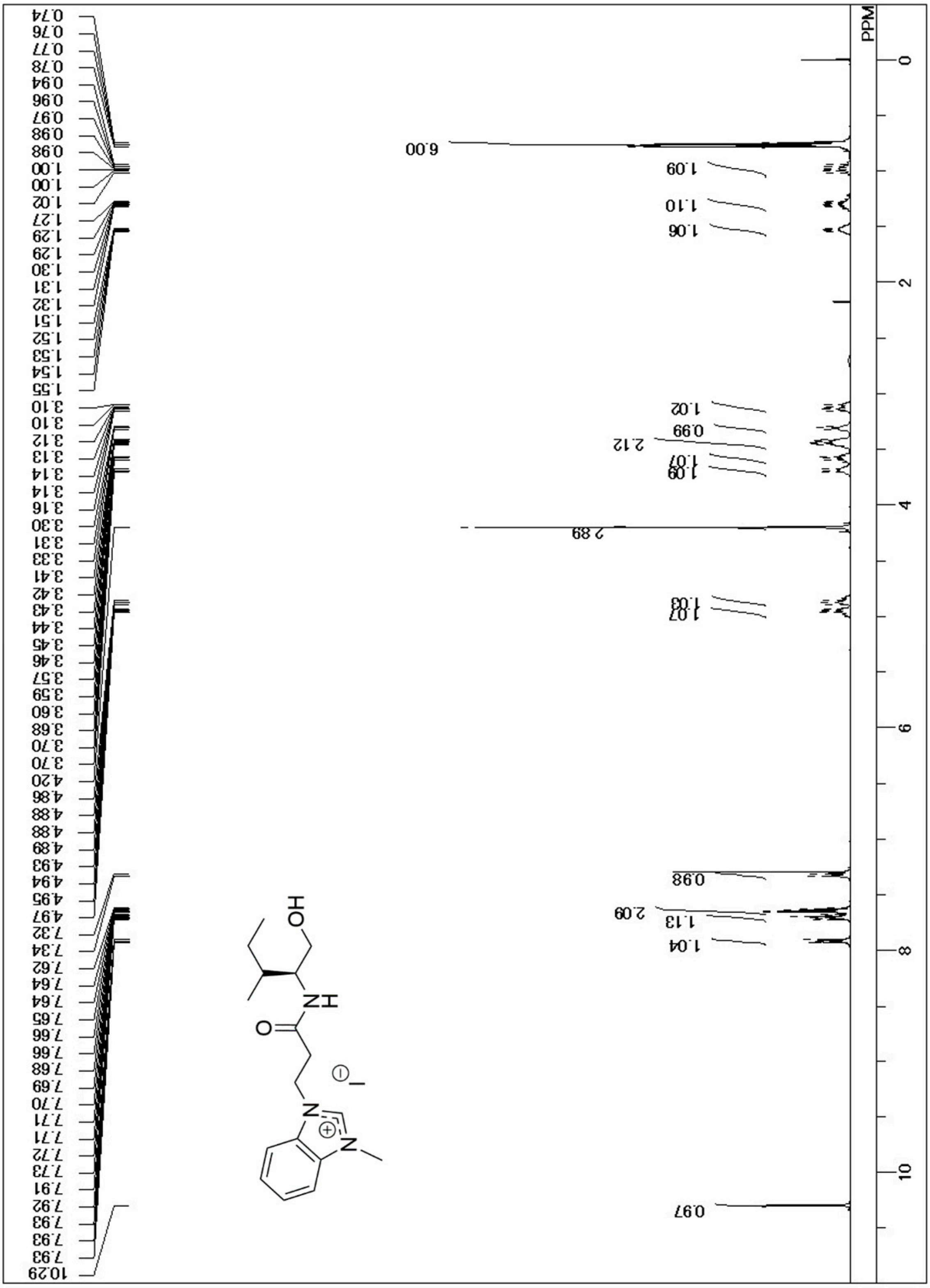

5d (R^1=^{s}Bu, R^2=Me).

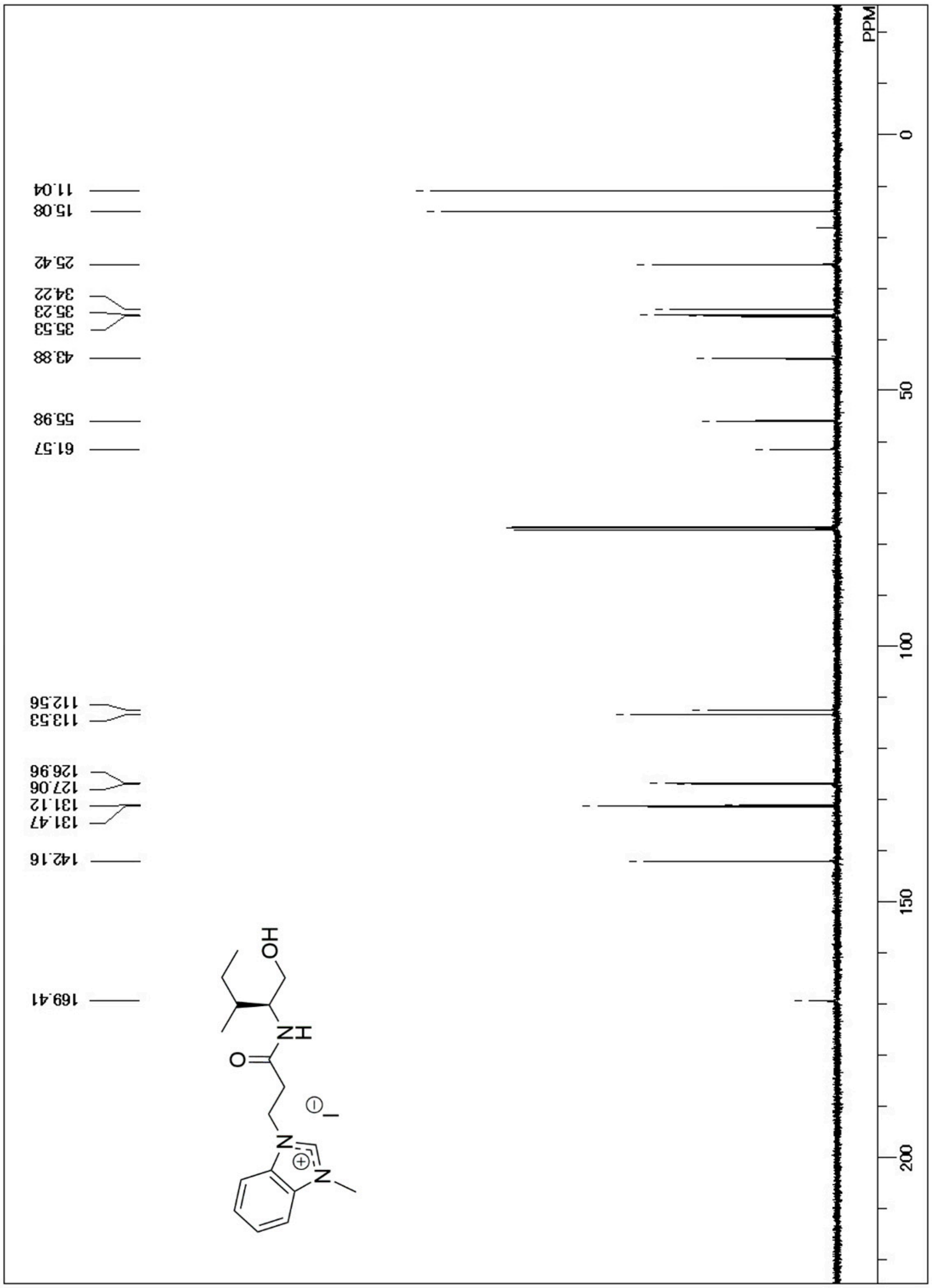

5d (R^1=^{s}Bu, R^2=Me).

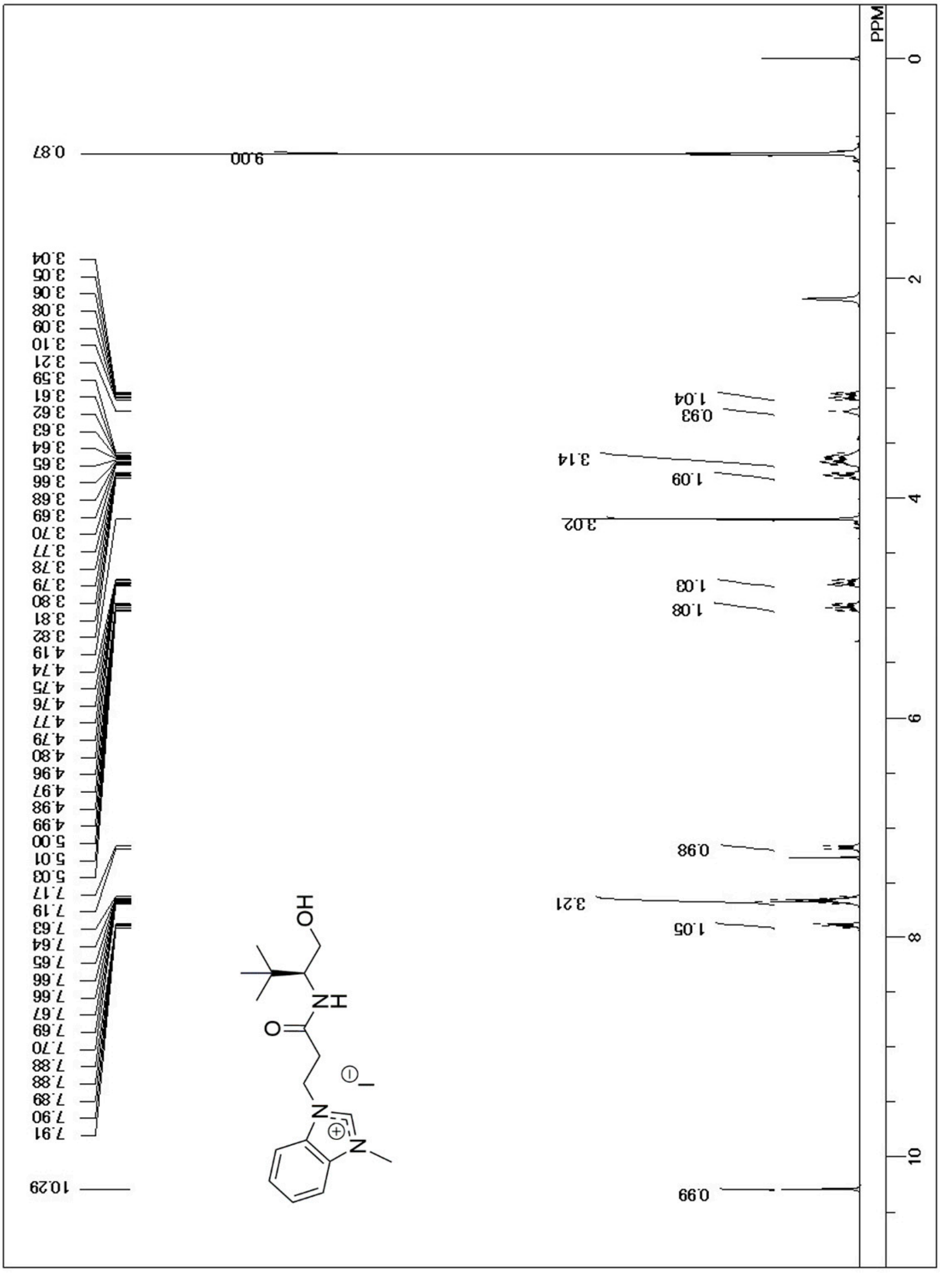

5e (R[1]=^{t}Bu, R[2]=Me).

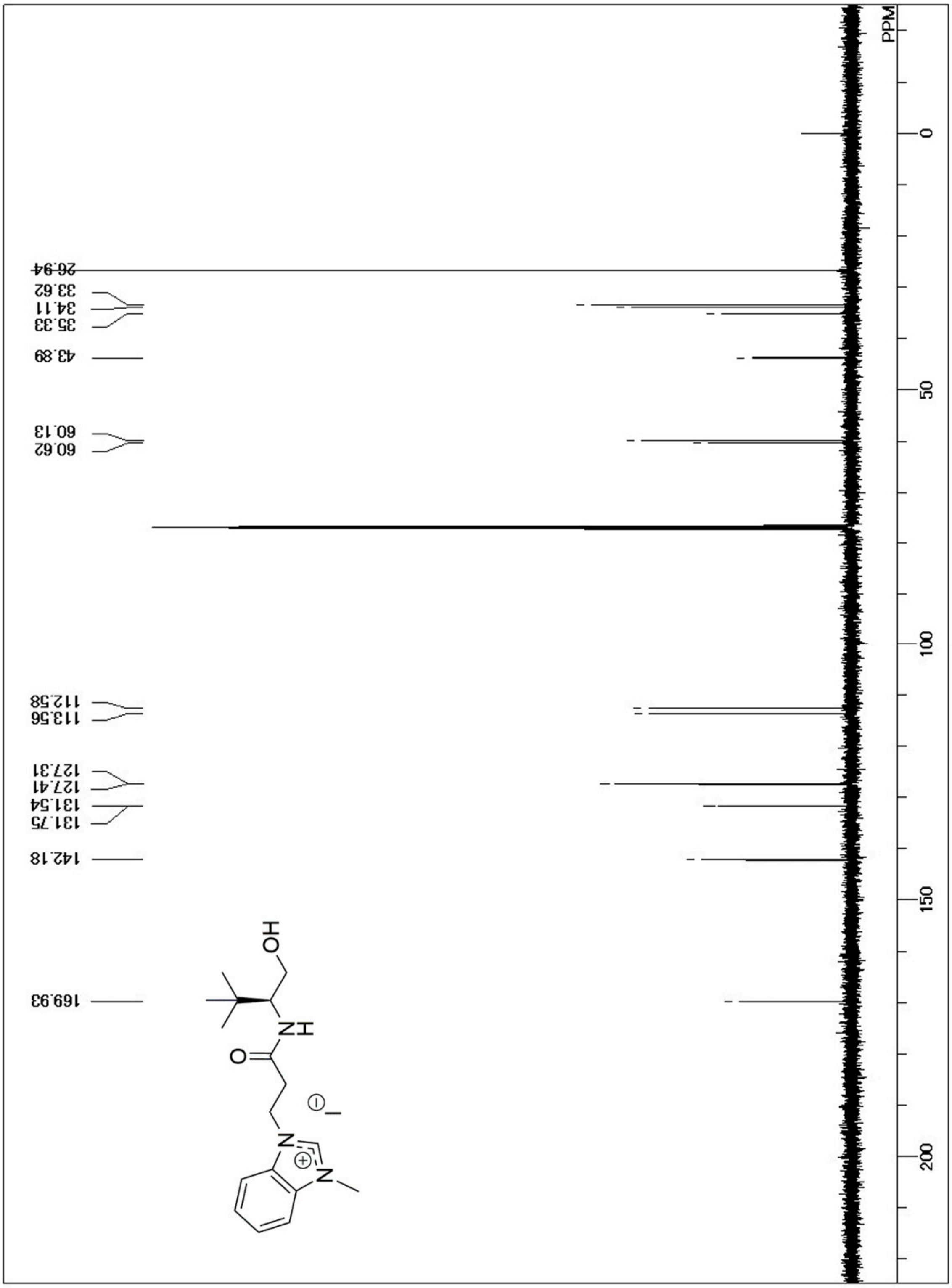

5e (R^1=^{t}Bu, R^2=Me).

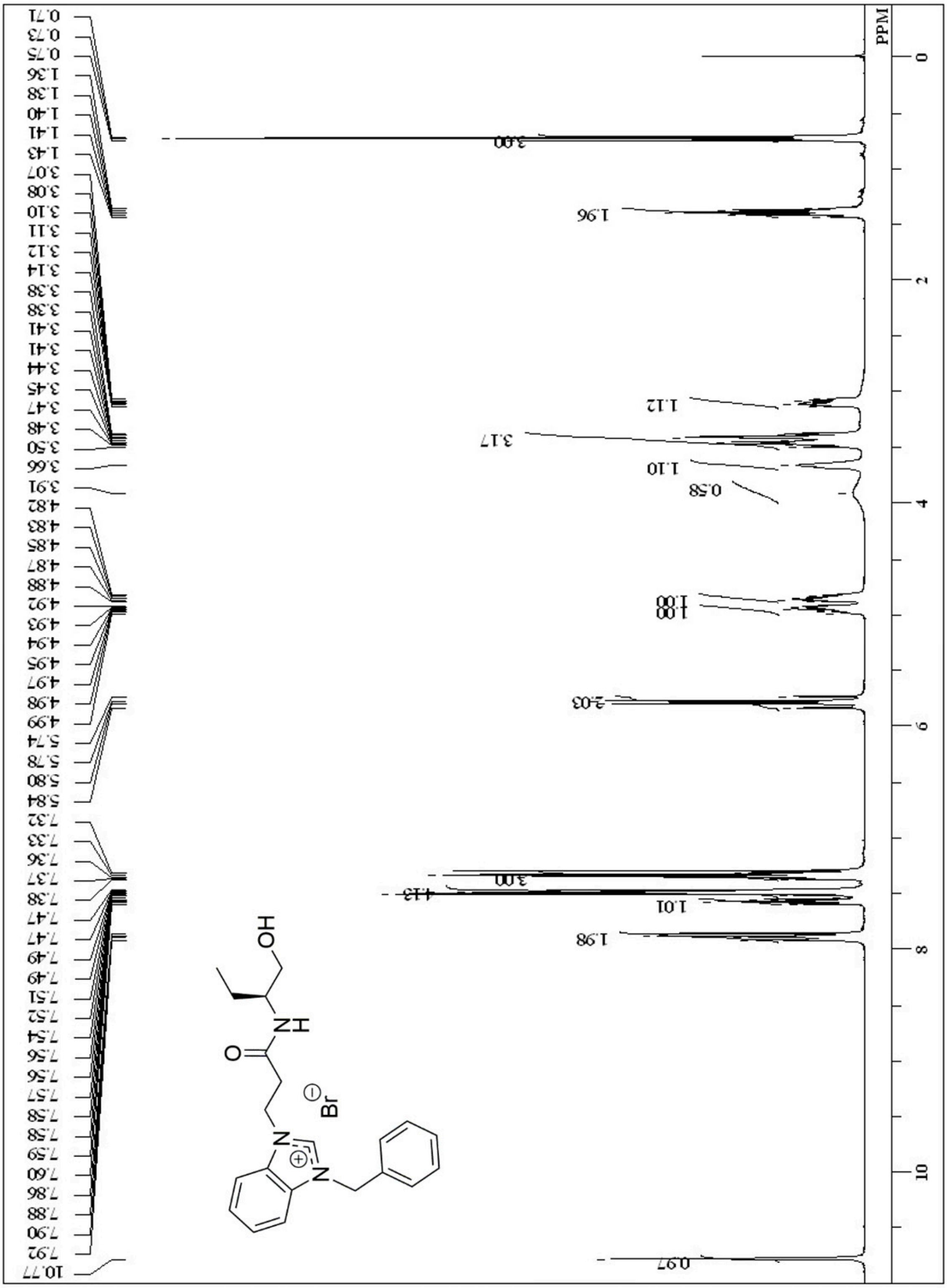

10b (R[1]=Et, R[2]=Bn).

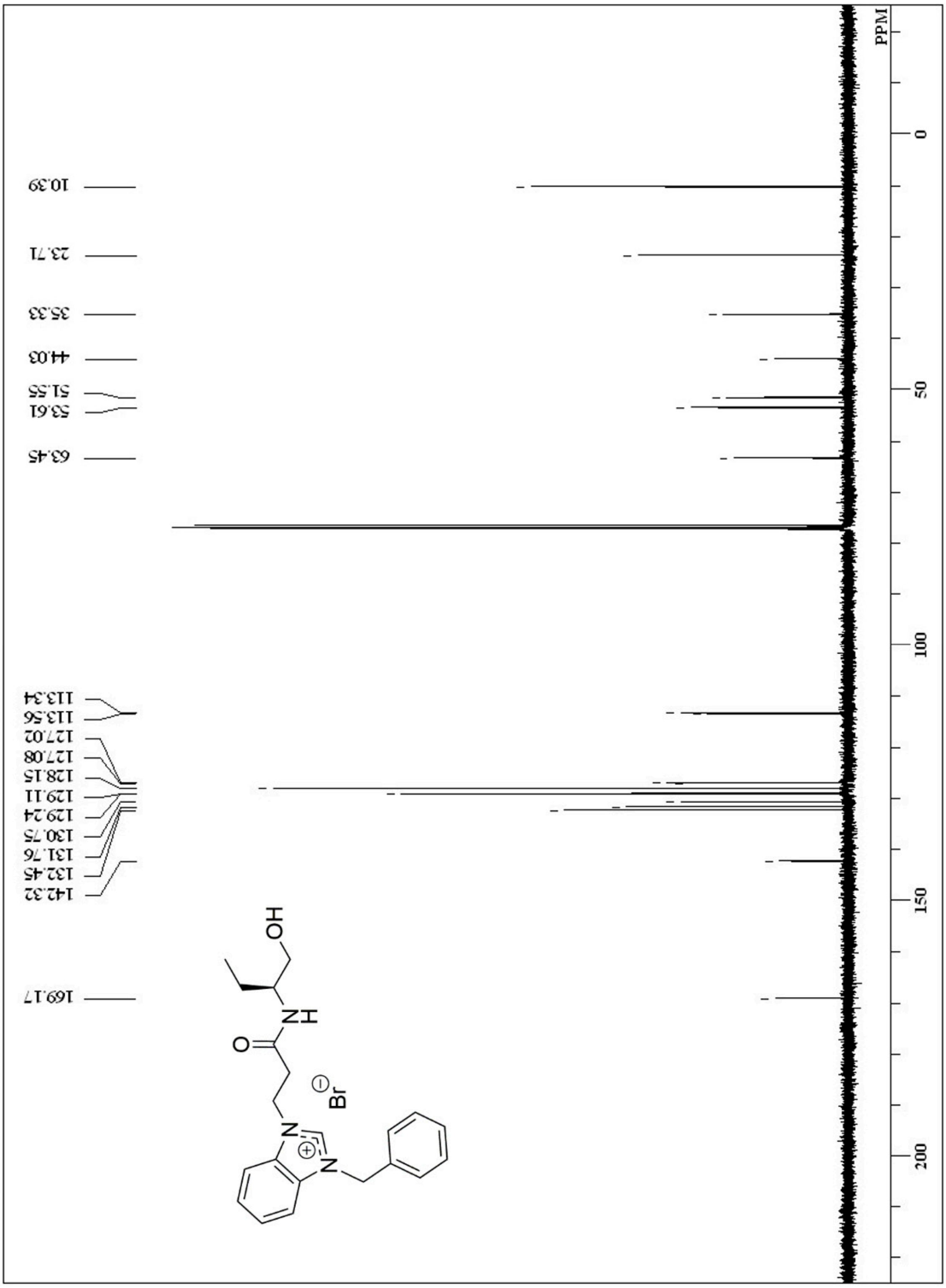

10b (R^1=Et, R^2=Bn).

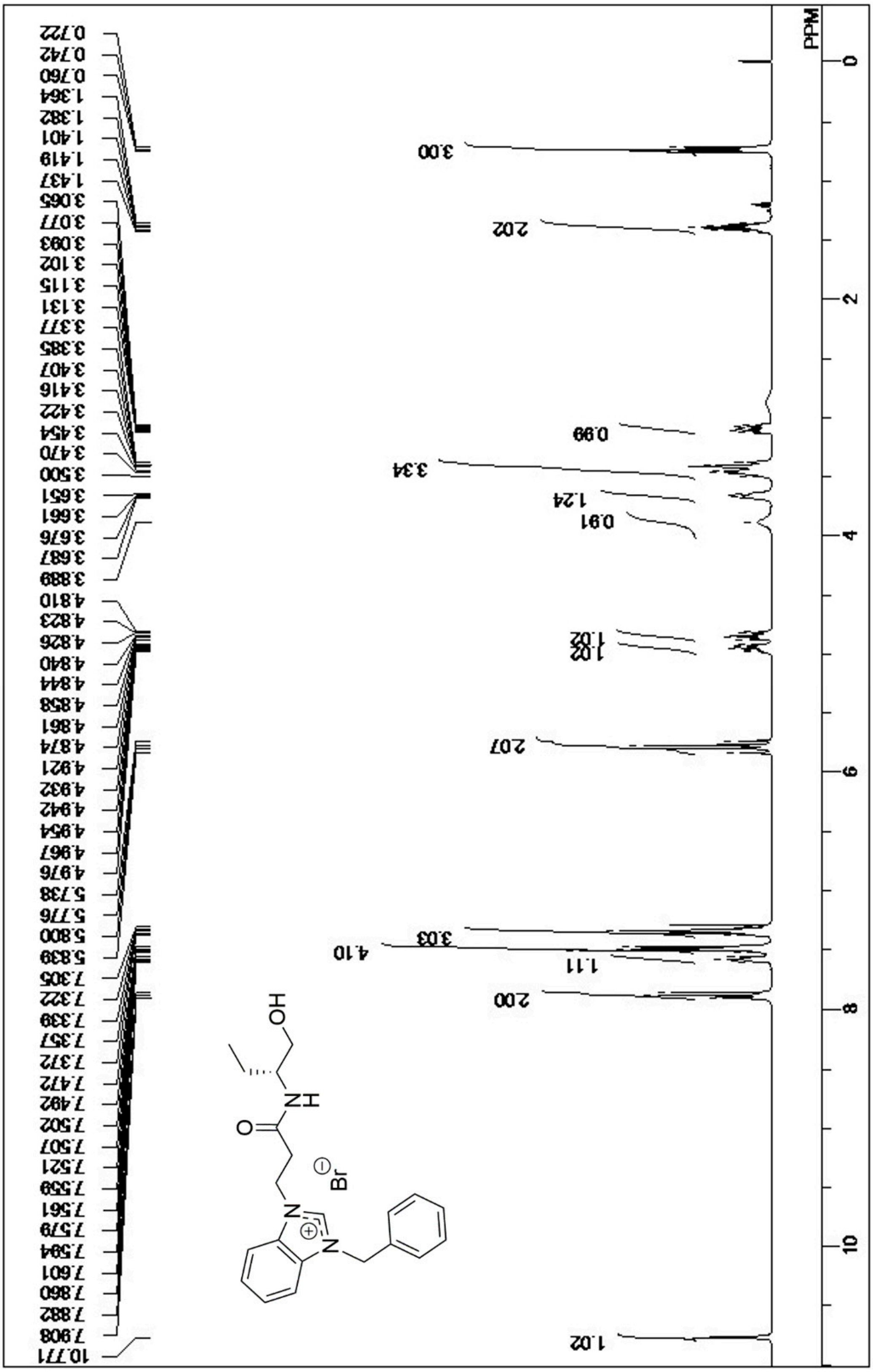

Ent-**10b (R[1]=Et, R[2]=Bn).**

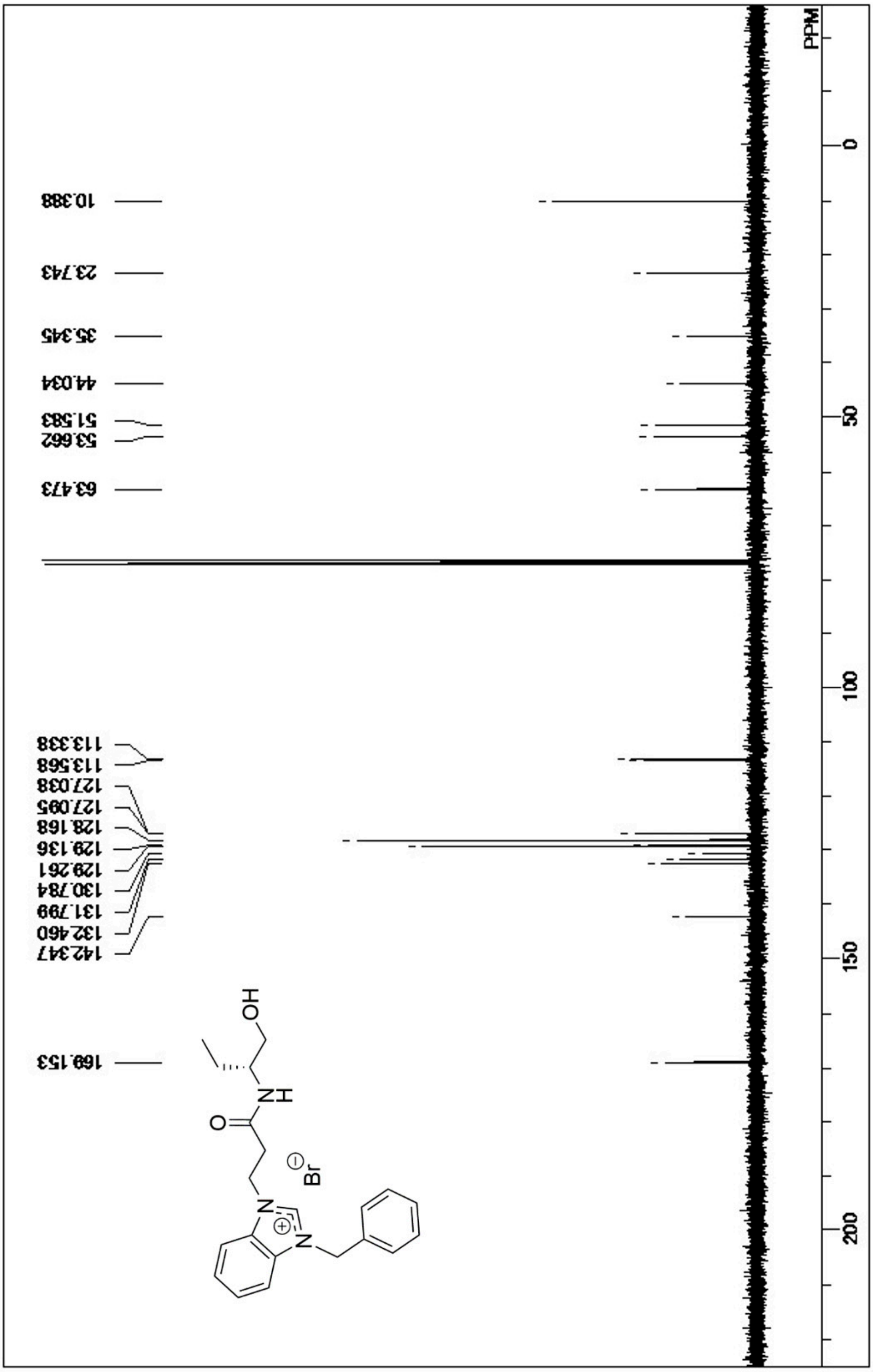

***Ent*-10b (R¹=Et, R²=Bn).**

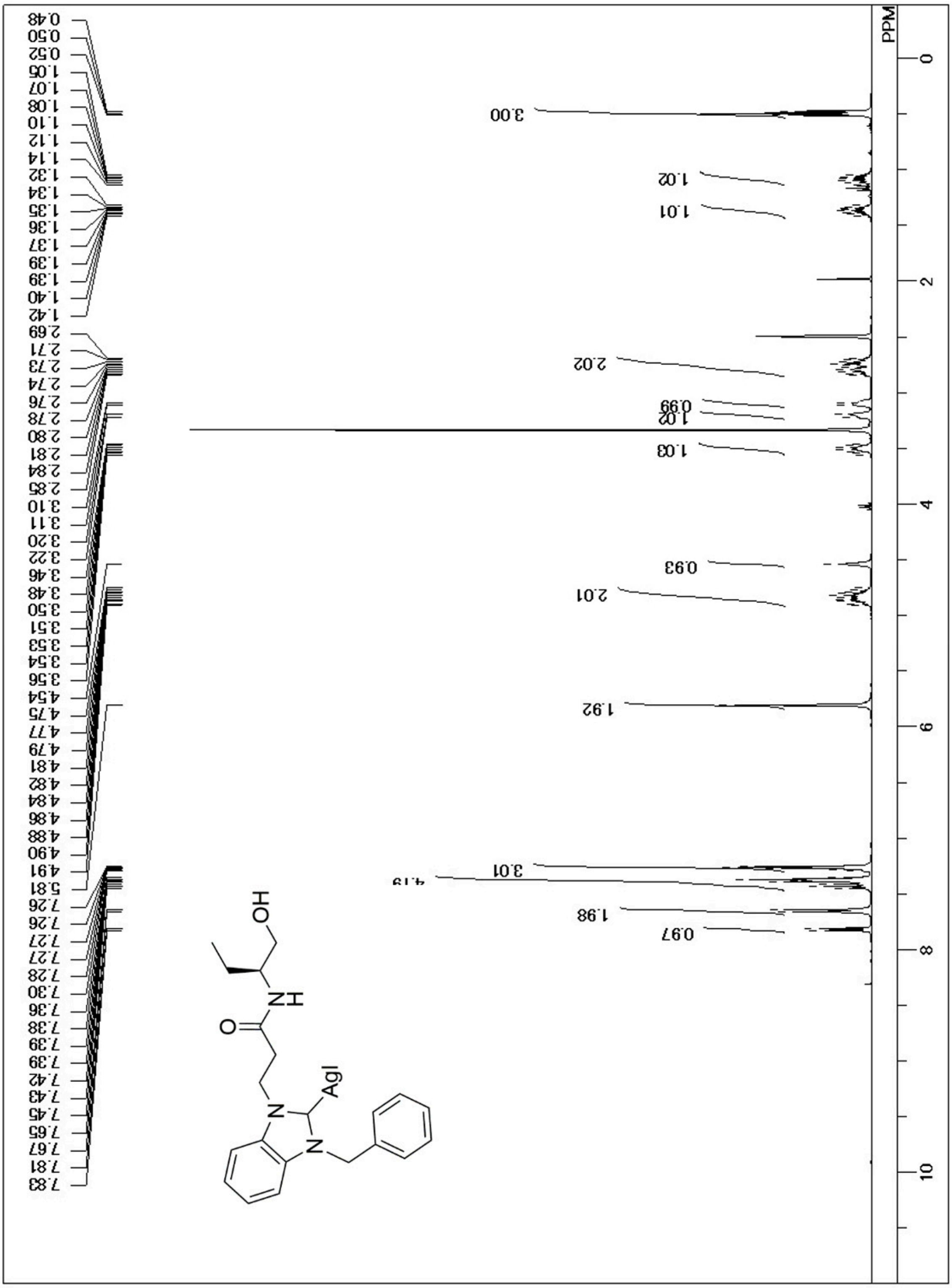

NHC•AgI 1b (R[1]=Et, R[2]=Bn).

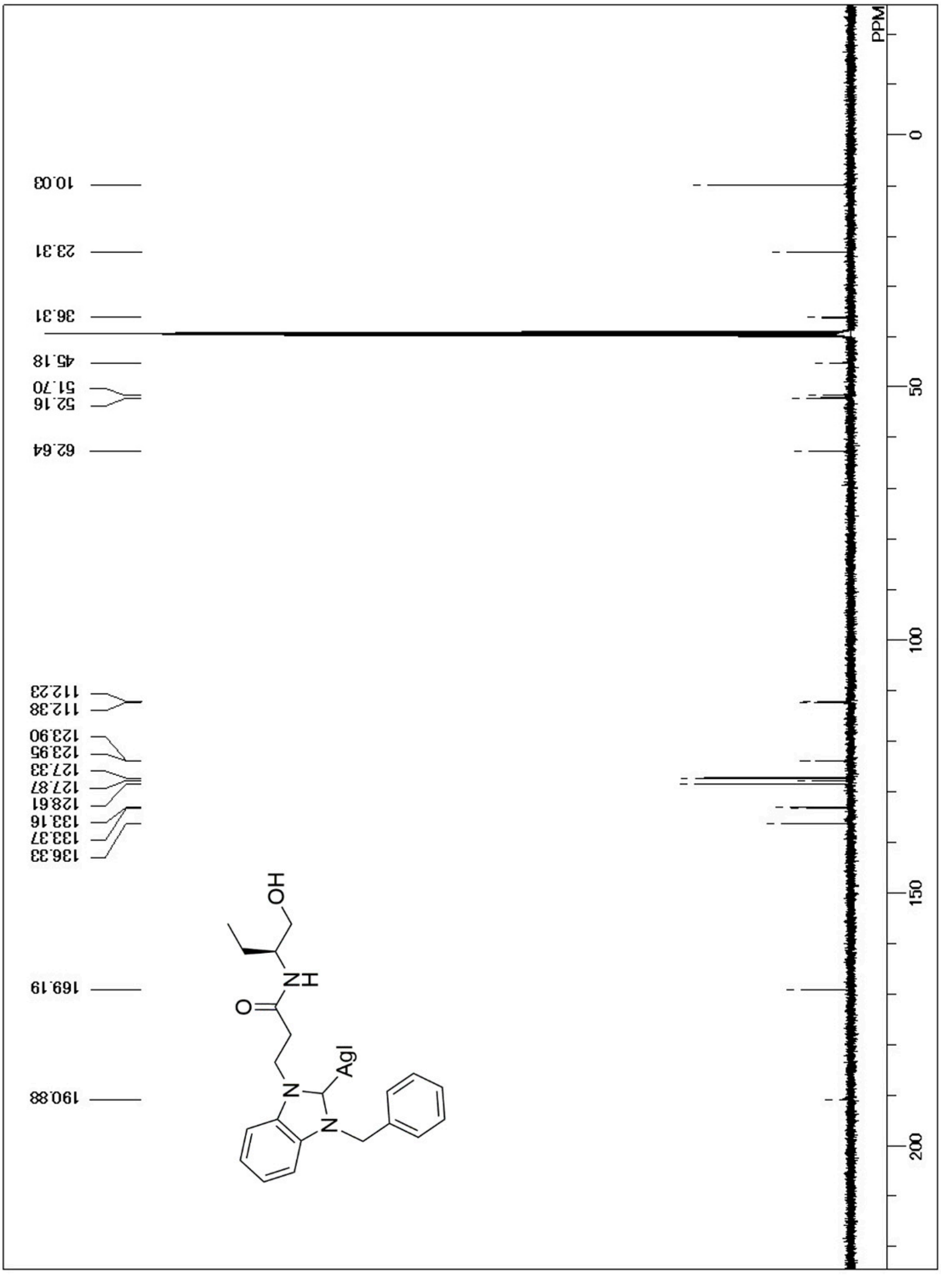

NHC • AgI 1b (R^1=Et, R^2=Bn).

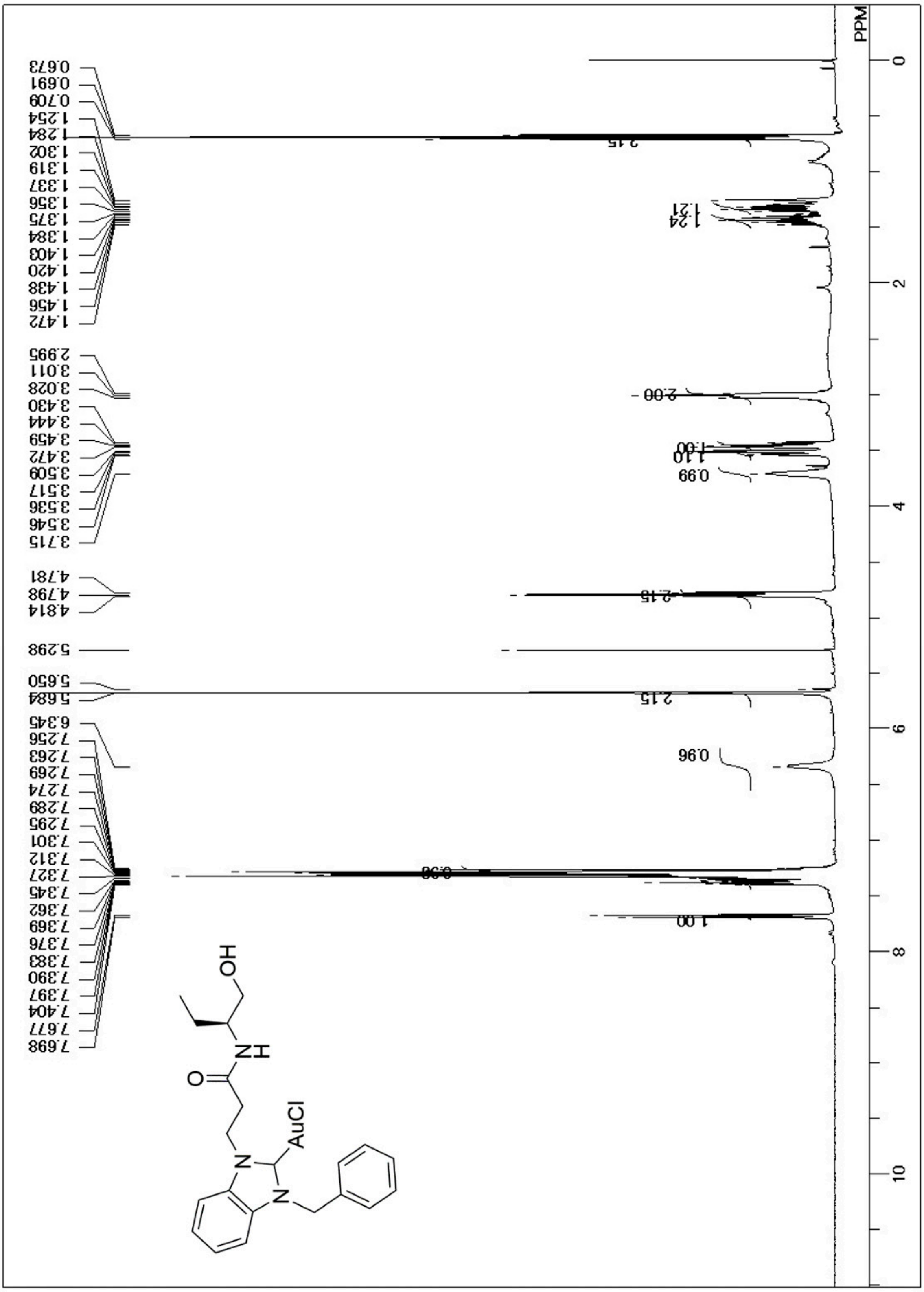

NHC • AuCl 11b (R^1=Et, R^2=Bn).

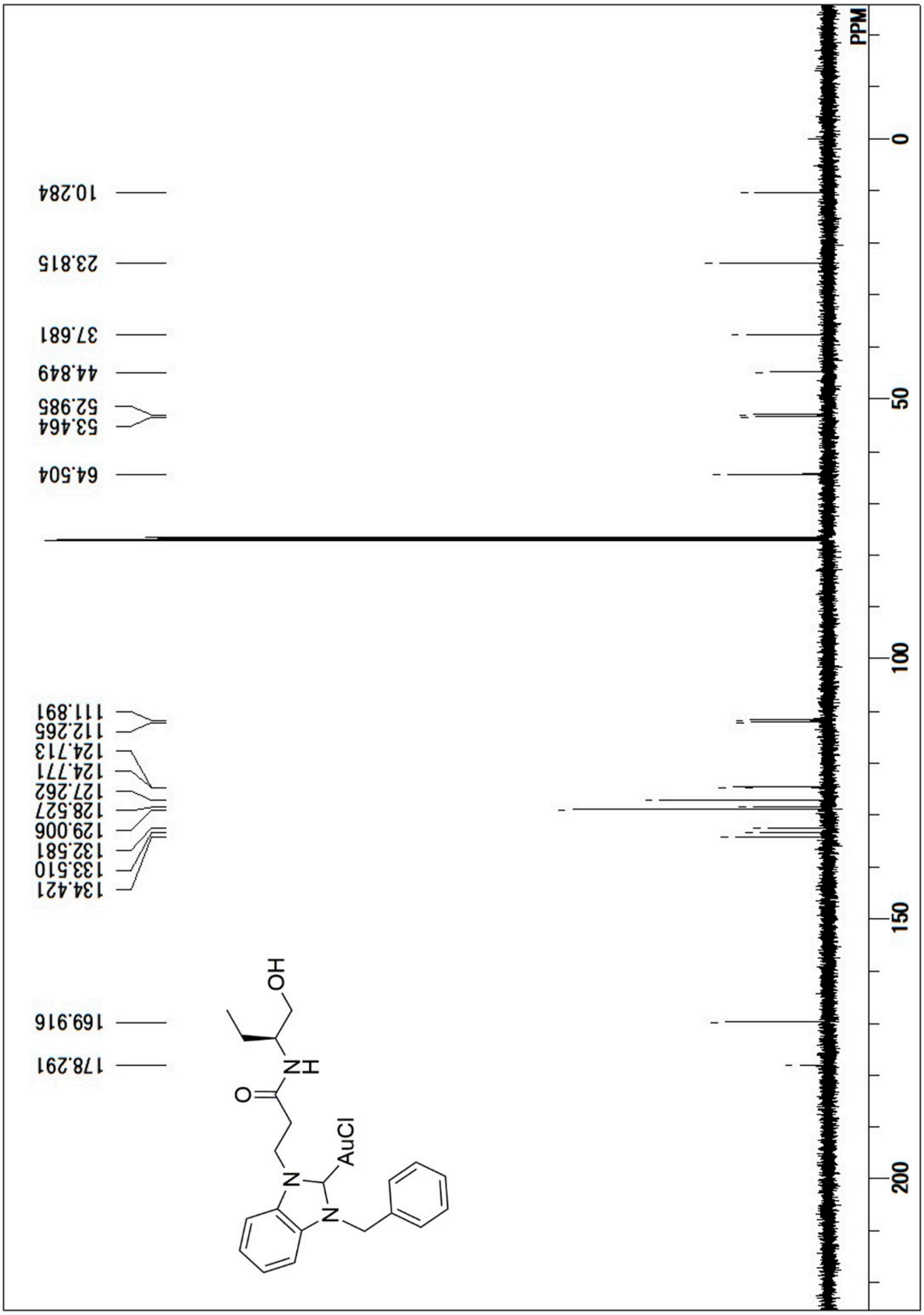

NHC • AuCl 11b (R[1]=Et, R[2]=Bn).

4. Selected Chiral GC Traces in the ACA Reaction

The enantiomeric excess of 1,4-adducts in the ACA reaction was determined by chiral GLC according to the previously reported procedures..

**3-Ethylcyclohexanone (3): pages 37-39**	Supelco γ-Dex225<br>70 °C, N_2 gas<br>Linear velocity of 27.5 cm/s	Rt = 58 min (*S*)<br>Rt = 60 min (*R*)
**3-Ethyl-4,4-dimethylcyclohexanone (6):** page 40	Supelco γ-Dex225,<br>85 °C, N_2 gas<br>Linear velocity of 27.5 cm/s	Rt = 58 min (*R*)<br>Rt = 59 min (*S*)
**3-Ethylcycloheptanone (7):** page 41	Supelco β-Dex225,<br>80 °C, N_2 gas<br>Linear velocity of 27.5 cm/s	Rt = 57 min (*S*)<br>Rt = 60 min (*R*)
**3-Methylcyclohexanone (8)**: page 42	Supelco γ-Dex225<br>60 °C, N_2 gas<br>Linear velocity of 27.5 cm/s	Rt = 53 min (*S*)<br>Rt = 60 min (*R*)
**3-Methylcycloheptanone (9)**: page 42	Supelco β-Dex225<br>70 °C, N_2 gas<br>Linear velocity of 27.5 cm/s	Rt = 58 min (*S*)<br>Rt = 72 min (*R*)

3-Ethylcyclohexanone (3)

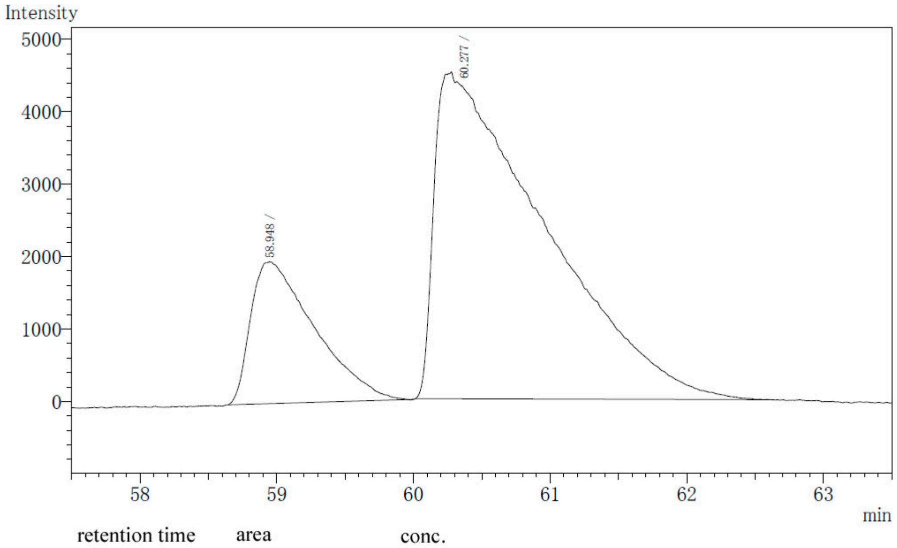

retention time	area	conc.
58.948	63402	20.095
60.277	252104	79.905

Table 1, entry 2, right: (*S*)-**3**; 80% *ee*

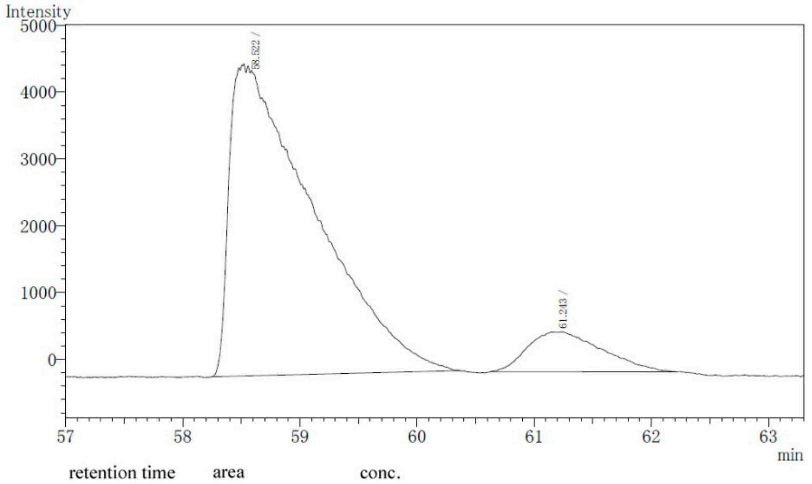

retention time	area	conc.
58.522	232473	89.758
61.243	26528	10.242

Table 2, entry 5, left: (*R*)-**3**; 75% *ee*

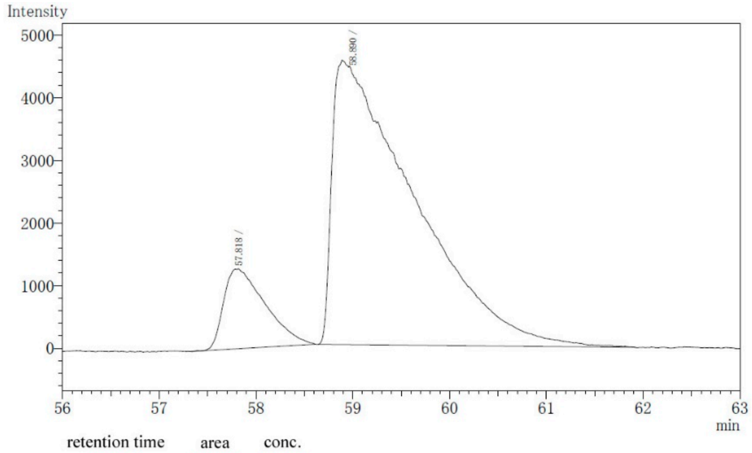

retention time	area	conc.
57.818	37973	12.462
58.890	266745	87.538

Table 2, entry 3, right: (*S*)-**3**; 87% *ee*

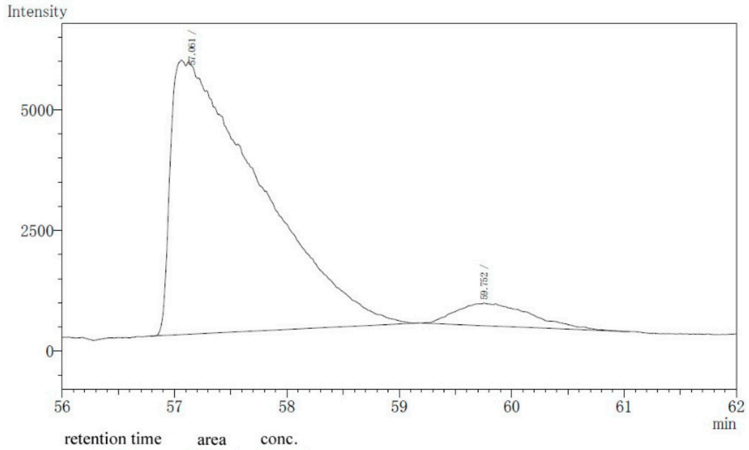

retention time	area	conc.
57.061	313453	93.494
59.752	21814	6.506

Table 2, entry 7, right: (*S*)-**3**; 89% *ee*

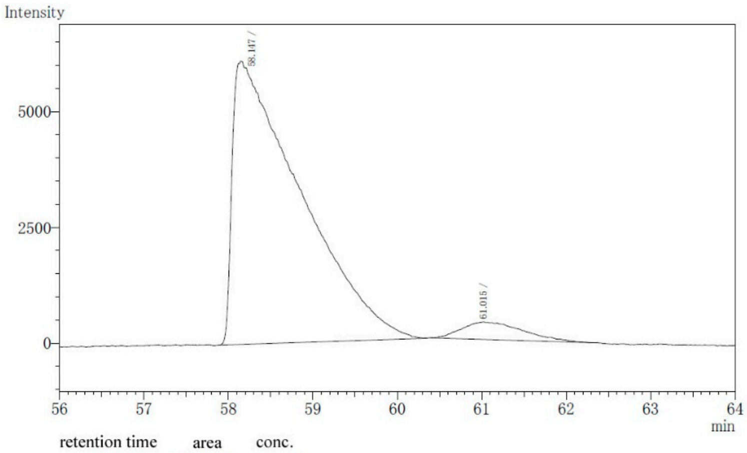

retention time	area	conc.
58.147	342427	94.688
61.015	19211	5.312

Table 3, entry 2, left: (*S*)-**3**; 69% *ee*

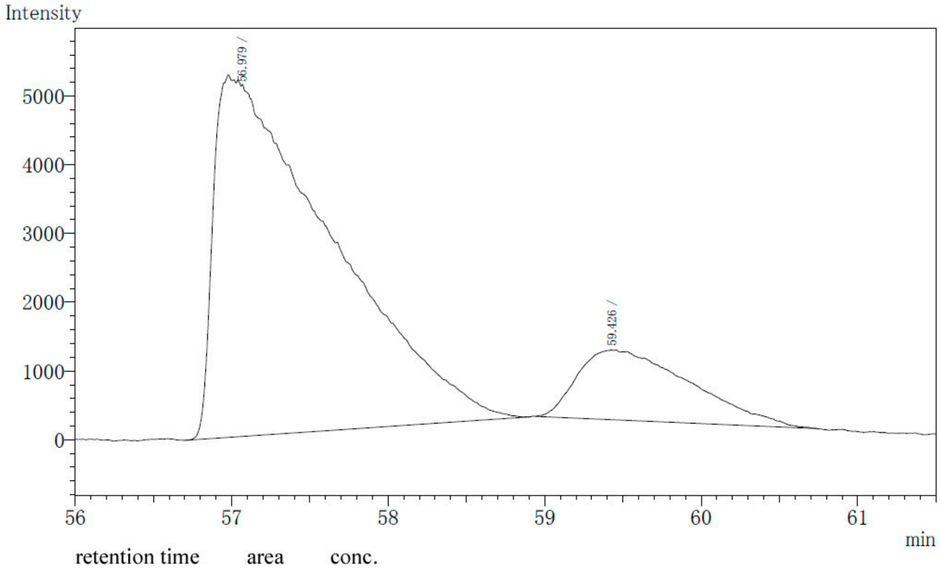

retention time	area	conc.
56.979	273979	84.264
59.426	51166	15.736

Table 3, entry 2, right: (*R*)-**3**; 78% *ee*

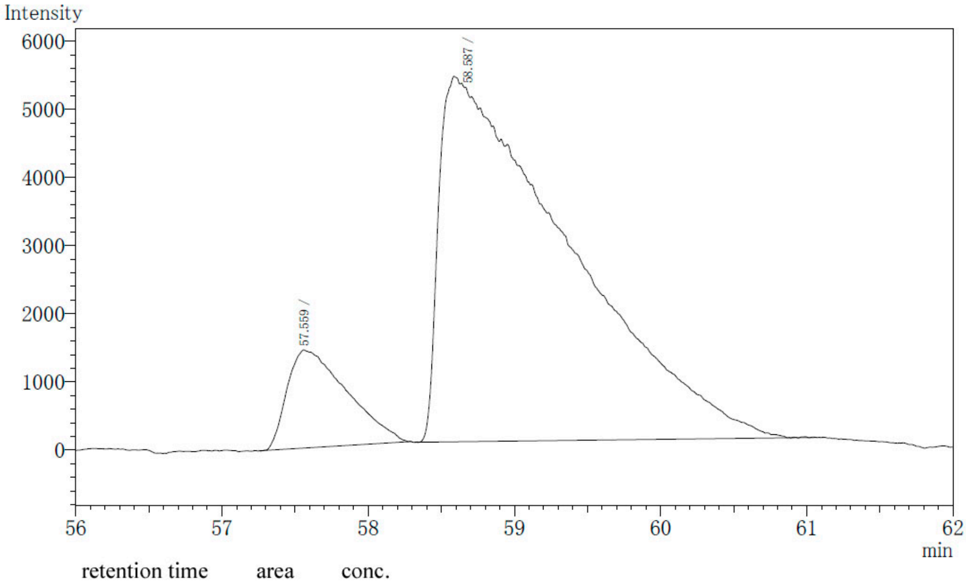

retention time	area	conc.
57.559	40676	10.956
58.587	330581	89.044

3-Ethyl-4,4-dimethylcyclohexanone (6)

Table 3, entry 3, left: (*S*)-**6**; 57% *ee*

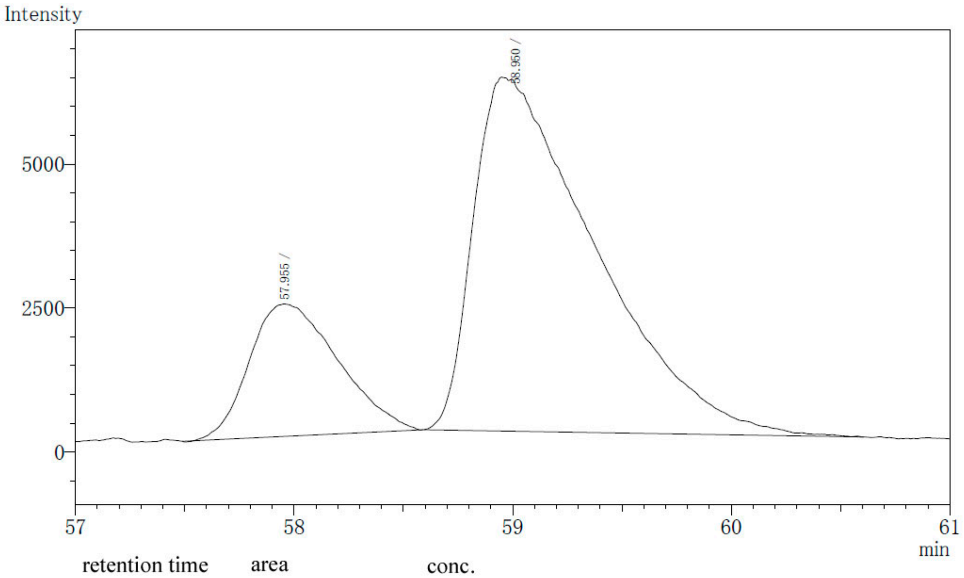

retention time	area	conc.
57.955	64684	21.378
58.950	237889	78.622

Table 3, entry 3, right: (*R*)-**6**; 84% *ee*

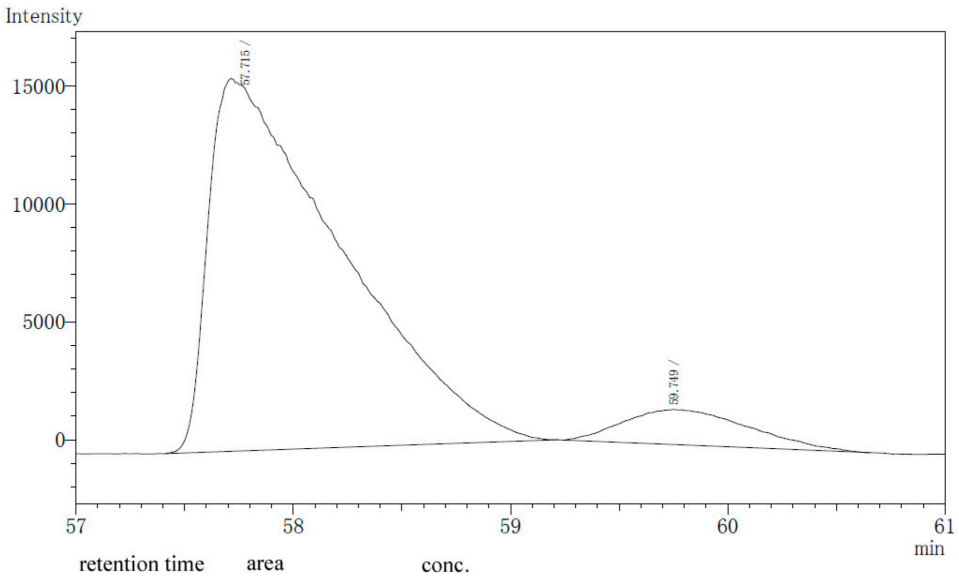

retention time	area	conc.
57.715	668616	91.772
59.749	59949	8.228

3-Ethylcycloheptanone (7)

Table 3, entry 4, left: (*R*)-**7**; 73% *ee*

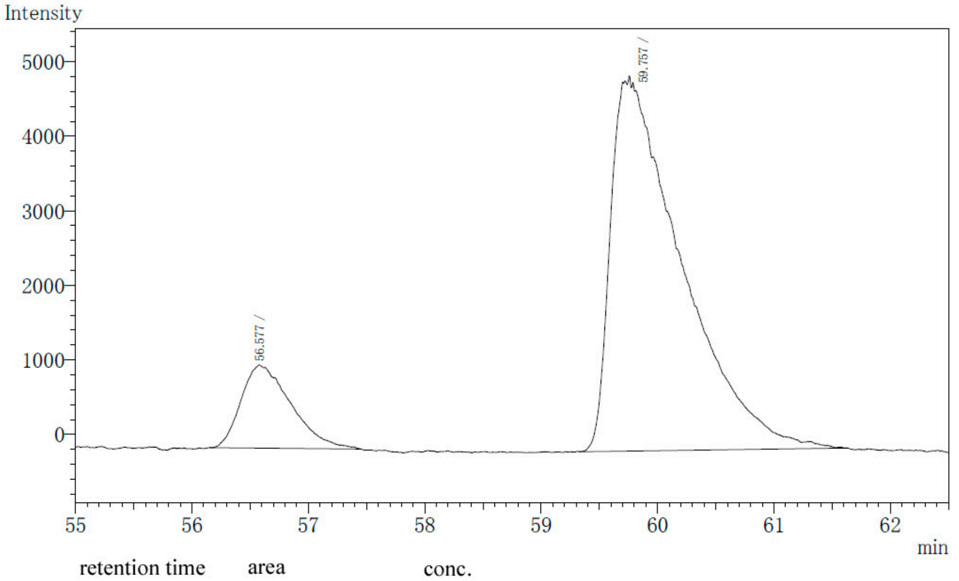

retention time	area	conc.
56.577	33183	13.520
59.757	212255	86.480

Table 3, entry 4, right: (*S*)-**7**; 81% *ee*

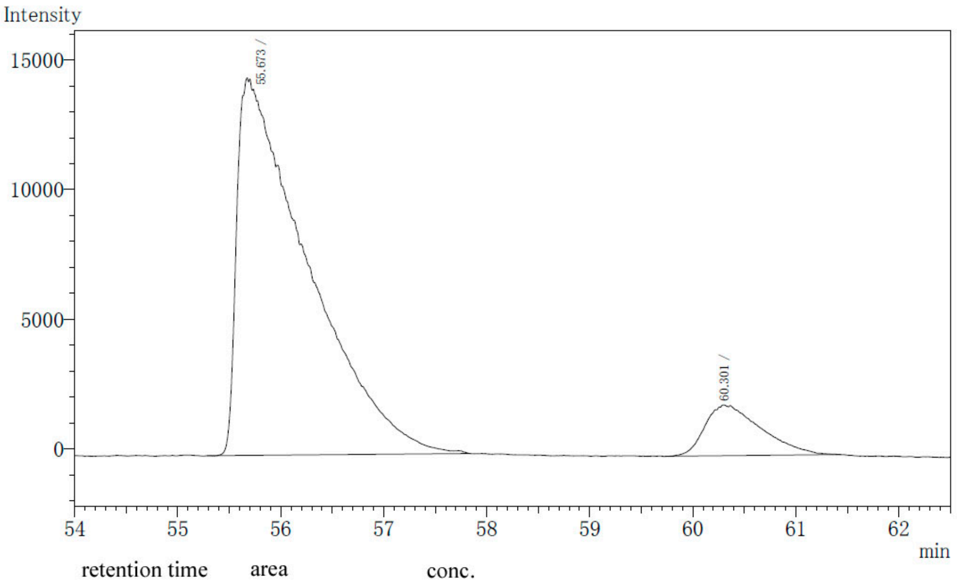

retention time	area	conc.
55.673	692217	90.346
60.301	73969	9.654

3-Methylcyclohexanone (8)
Table 3, entry 5, right: (*S*)-**8**; 93% *ee*

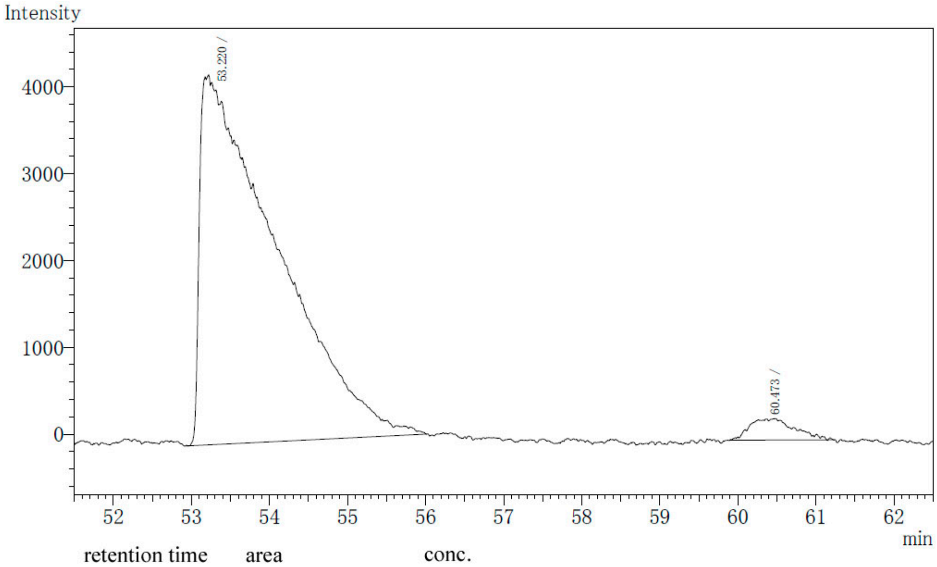

retention time	area	conc.
53.220	289259	96.684
60.473	9920	3.316

3-Methylcycloheptanone (9)
Table 3, entry 6, right: (*S*)-**9**; 93% *ee*

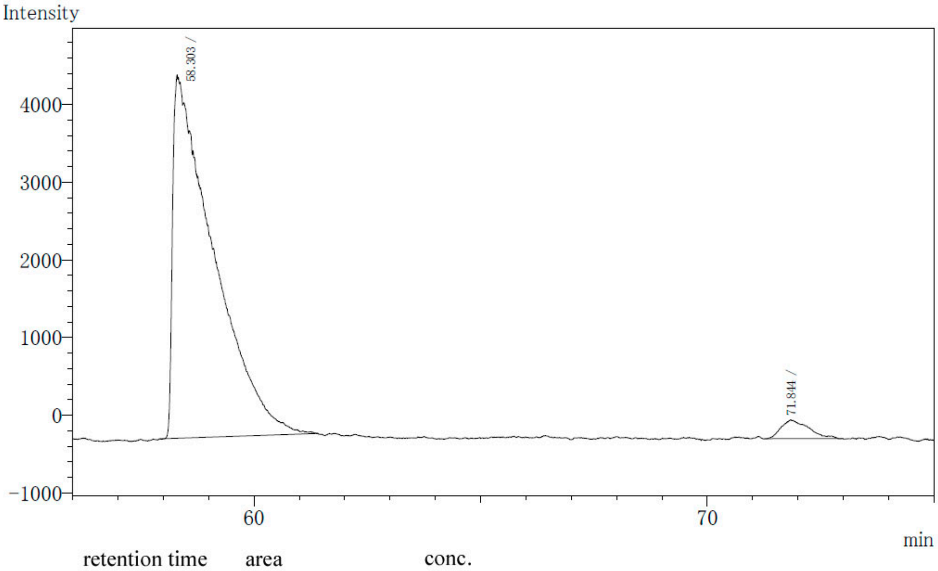

retention time	area	conc.
58.303	291486	96.633
71.844	10155	3.367
